# Impacts of anthropogenic and environmental stressors on biotic communities in Al-Mahmoudia Canal, Egypt: a seasonal assessment of water quality and plankton dynamics

**DOI:** 10.1038/s41598-026-62334-4

**Published:** 2026-07-27

**Authors:** Ahmed M. M. Heneash, Ahmed S. Shehata, Hazem T. Abd El-Hamid, Mohamed A. M. Heneash, Mohamed M. M. El-Feky

**Affiliations:** 1 National Institute of Oceanography and Fisheries, NIOF, Cairo, Egypt; 2 Marine Biotechnology and Natural Products Extraction (MBNPE) Lab., Marine Environment Division, National Institute of Oceanography and Fisheries, NIOF, Cairo, Egypt; 3 Environment and Natural Materials Research Institute (ENMRI), City of Scientific Research and Technological Applications (SRTA-City), New Borg El-Arab City, Alexandria 21934 Egypt; 4 Faculity of science, Al Azhar University, Cairo, Egypt; 5 Natural Resources Studies and Research Department, College of High Asian Studies, Zgazig University, Zagazig, 44519 Egypt

**Keywords:** Al-Mahmoudia Canal, Water quality, Heavy metals, Seasonal variation, Zooplankton, Microbial contamination, Cluster analysis, Ecology, Ecology, Environmental sciences, Microbiology

## Abstract

Al-Mahmoudia Canal is crucial for water supply in Egypt’s Al-Beheira and Alexandria governorates, but it faces ecological challenges from pollution. This study examines plankton dynamics and water quality across four seasons at seven sites, analyzing microbiological contamination, heavy metals, turbidity, and nutrients. Phytoplankton abundance peaked in spring at Station 4, with seasonal variations linked to temperature and nutrients. Zooplankton showed seasonal diversity, with increased turbidity and nutrient levels in summer and autumn due to organic pollution. Heavy metal and microbial contamination also fluctuated seasonally, with fecal coliform levels exceeding safety limits. The study highlights pollution hotspots and emphasizes the need for ongoing monitoring and management to ensure sustainable water resource use in the Nile Delta.

**Supplementary Information:**

The online version contains supplementary material available at 10.1038/s41598-026-62334-4.

## Introduction

Studies on El-Mahmoudia Canal, an essential water supply for the Egyptian governorates of Alexandria and Beheira, supporting agriculture, industry, and domestic use, reveal significant water quality and ecological concerns. Water Quality Index assessments consistently range from 75.01 to 132.57, indicating persistently poor to very bad conditions^1,2^. Nutrient enrichment and organic pollution are indicated by seasonal fluctuations in the canal’s physicochemical parameters, which include temperature, dissolved oxygen, nutrients, and chlorophyll-a concentrations^2^. Rotifers predominate in zooplankton groups and are biomarkers of eutrophic environments^3,4^. Rotifer abundance exhibits seasonal trends, peaking at 49,000 organisms/m^3^ in the fall and falling to 24,500 organisms/m^3^ in the spring^4^. The zooplankton community structure, combined with water quality parameters, provides an effective tool for assessing the ecological health of Egyptian freshwater canals, revealing the canal’s compromised environmental status due to pollution pressures. Zooplankton composition further reflected these environmental gradients. Crustaceans dominated the Mediterranean Sea, emphasizing their central role in marine food-web dynamics^5^. Zooplankton plays a crucial role in aquatic ecosystems by serving as a key link in energy transfer from primary producers (phytoplankton) to higher trophic levels, including fish, marine mammals, and turtles. As preferred prey for many aquatic organisms, their abundance and diversity directly influence the health and stability of food webs^6–10^. Monitoring zooplankton populations provides valuable insights into ecosystem productivity, water quality, and the overall functioning of marine and freshwater environments (Richardson 2008)^11–14^ The construction of the Aswan High Dam and subsequent abrupt curtailment of the seasonal outflow of nutrient-rich Nile water towards the sea caused dramatic changes in the biological and physico-chemical characteristics of the southeast Mediterranean environment off the Egyptian coast^6,8–10,15^. Agricultural water streams in Egypt are increasingly contaminated by diverse pollutants from agricultural, domestic, industrial, and municipal sources. Organic and inorganic pollutants-including pesticides, plasticizers, and plant residues have been detected in these water bodies. Key water quality parameters such as turbidity, BOD, COD, NH_4_, HCO_3_, MPN, and Cu frequently exceed safety thresholds set by the Egyptian Ministry of Irrigation, WHO, and FAO guidelines. This contamination is exacerbated by the mixing of domestic and industrial wastewater with agricultural drainage. In Upper Egypt, all drains ultimately discharge back into the Nile River, leading to widespread pollution of irrigation canals, including the El-Mahmoudia Canal. Given the seasonal variability in water quality, this study aims to assess and characterize the types and concentrations of pollutants including heavy metals and organic contaminants in the El-Mahmoudia Canal across winter, spring, summer, and autumn seasons^16,17^.

This study investigates the impacts of abiotic factors influenced by climate change and anthropogenic activities, along with Nile River discharge dynamics, on the planktonic community structure in Al-Mahmoudia Canal. The research specifically examines how seasonal variations in physicochemical parameters (temperature, salinity, nutrient loading) and hydrological modifications affect phytoplankton and zooplankton composition, abundance, and diversity. Particular emphasis is placed on understanding the synergistic effects of climate-driven changes and human-induced stressors on this critical freshwater ecosystem.

The uniqueness of the current research employs an ecosystem-based approach to assess the ecological state of Al-Mahmoudia Canal, focusing on water quality, heavy metals, microbiological contamination, phytoplankton, and zooplankton. It also analyzes the relationship between environmental factors and plankton community dynamics, providing baseline data to support future monitoring and sustainable management of this important freshwater canal in Egypt.

## Materials and methods

Al-Mahmoudia Canal is strategically located in northwestern Egypt, forming the northern boundary of Beheira Governorate. This vital waterway originates from the Rosetta Branch of the Nile River at Mahmoudia City (km 194.200) and serves as a crucial irrigation source for approximately 130,200 hectares of agricultural land. With an average daily water discharge of 15 million cubic meters, the canal plays a pivotal role in the region’s agricultural economy and water management system (Abo Kila 2012). Al-Mahmoudia Canal extends 77.170 km and supplies water to seventy subsidiary canals, serving as a critical irrigation artery in northwestern Egypt. The canal derives water from three primary sources: two freshwater sources, which are from the Rosetta branch via the El-Atf pump station at the canal’s head and the Al-Khandaq Eastern canal at km 13.200; and the third is drainage water from the Zarkon Drain (km 8.500), lifted into Al-Mahmoudia via the Edko pump station. In addition to these regulated inflows, the canal receives non-point source pollution, including agricultural runoff and domestic wastewater, primarily through the Zarkon Drain. These mixed inputs influence the canal’s water quality, necessitating ongoing monitoring to balance agricultural demand and ecological health^2^.

### Sampling, measurements and analysis

**Fig. 1:**
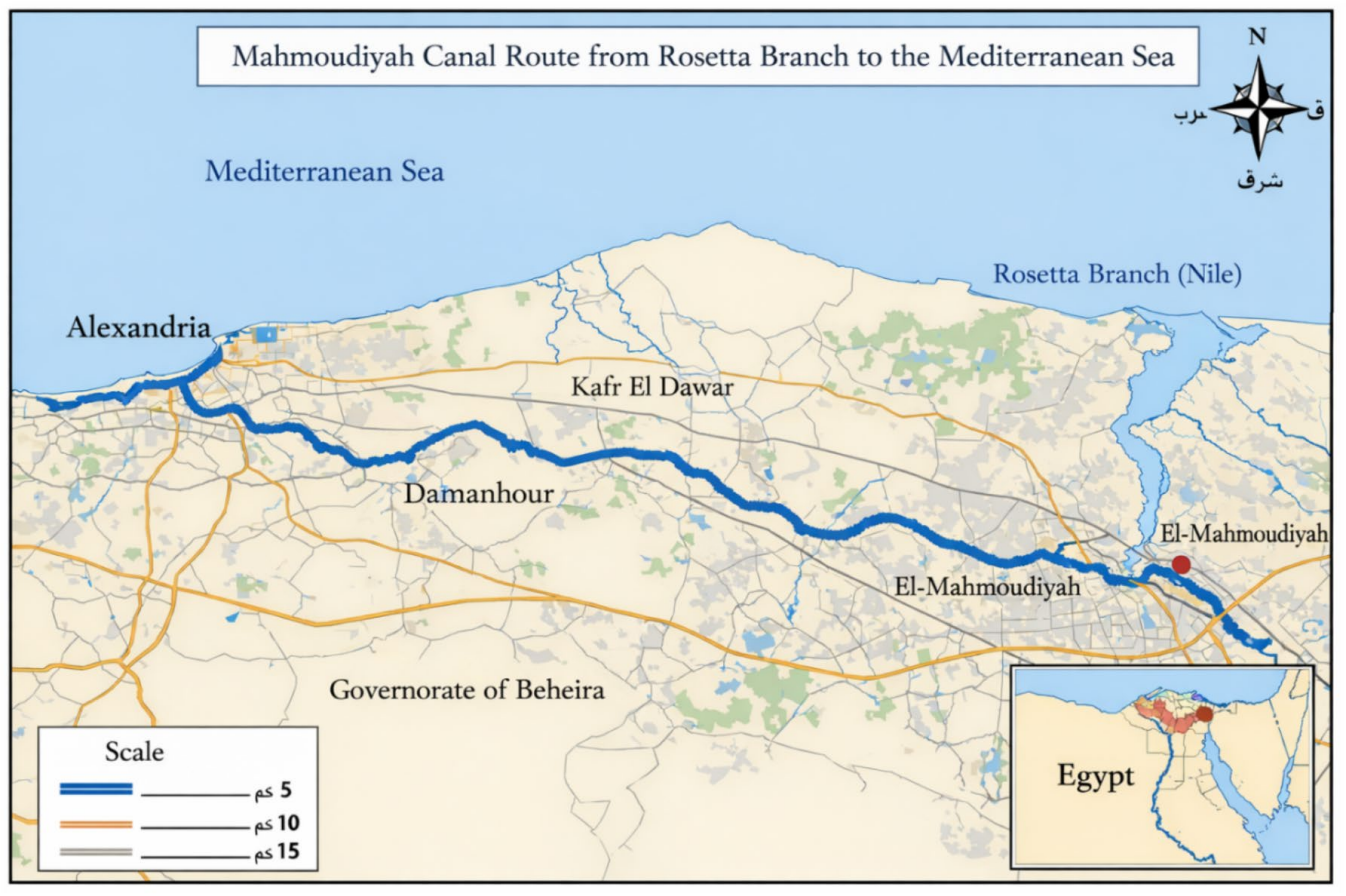
Al-Mahmoudia Canal location map and sampling locations

For this study, water samples were methodically collected across four seasons (winter, spring, summer, and autumn) during 2023 to capture temporal variations in water quality. Seven strategically selected sampling stations (Mahmodia Canal, Zarkon, End of Khandak Canal, Town of Abou Hommos, Town of Kafr El Dawar, Khorshid intake, Manshia WTP intake) were established along Al-Mahmoudia Canal, representing distinct environmental characteristics and anthropogenic influences along the watercourse. The geographical distribution and specific features of these monitoring stations are illustrated in Fig. 1. The stratified sampling approach involves selecting stations that represent variations in water sources, land use patterns, anthropogenic pressures, and hydrological gradients along the canal. This method ensures comprehensive spatial coverage and enables seasonal comparisons of water quality parameters throughout the year.

### Physicochemical analysis

Water quality assessment was conducted through comprehensive analysis of physical, chemical, and microbiological parameters, with seasonal measurements documented in Table 1. Field measurements, temperature and pH were determined in situ immediately following sample collection. Laboratory analyses were performed using standard methods in accordance with APHA (American Public Health Association) guidelines^18^, standard methods for the examination of water and wastewater, and relevant EPA (Environmental Protection Agency) protocols. The analytical approach encompassed physical parameters (e.g., turbidity, electrical conductivity), chemical characteristics (e.g., nutrients, dissolved oxygen, heavy metals); and microbiological indicators (e.g., total coliforms, faecal bacteria). This methodological framework ensures data quality and comparability with established water quality standards and previous research findings^18^.

**Table 1:** Seasonal variations of physico-chemical parameters (water quality) recorded at all sites at Al-Mahmoudia Canal

		Mahmodia Canal	Zarkon	End of Khandak Canal	Town of Abou Hommos	Town of Kafr El Dawar	Khorshid intake	Manshia WTP intake	Mahmodia Canal	Zarkon	End of Khandak Canal	Town of Abou Hommos	Town of Kafr El Dawar	Khorshid intake	Manshia WTP intake
		Summer							Autumn						
Inorganic Analysis		1	2	3	4	5	6	7	1	2	3	4	5	6	7
Turbidity	NTU	29.15	29.15	29.5	12	11.795	17.17	9.595	12.72	9.905	14.5	14.9	11.785	13.32	14.55
pH value	pH unit	7.935	7.955	7.83	7.885	7.94	7.935	7.865	7.805	7.7	7.77	7.785	7.77	7.715	7.885
E.C	µS/cm	640	343	627	411.5	468.5	453.5	420.5	603	378.5	608	338	411	1005	406
Hardness, total	mg/L	193.5	114.5	203.5	128.5	122.5	124	121	227.5	134.5	224	122	141.5	199	157
Alkalinity, total	mg/L	210	156	217	159	154	149	155	209	143	199	140	151	207	141
Chloride	mg/L	49.9	22.3	49	37	36	40.5	37	49.9	20.5	53.5	24.5	28.2	152	46.5
Phosphate	mg/L	0.085	0.02	0.145	0.09	0.125	0.12	0.085	0.16	0.07	0.16	0.05	0.07	0.085	0.075
Nitrate	mg/L	8.15	3.85	7.65	5.87	5.955	5.105	4.295	12.1	5.2	12.7	4.29	6.3	18.9	7.1
Nitrite	mg/L	0.29	0.555	0.315	1.04	0.125	0.145	0.095	0.535	0.09	0.531	0.082	0.15	0.256	0.086
Silica	mg/L	3.1	2.315	3.17	2.7	3.2	3.3	2.45	13.7	8.88	12.32	6.8	5.45	8.6	8.7
Sulphate	mg/L	77	22.4	71	89.9	127.45	131.1	38.6	65.5	30.1	71.1	32.3	43.75	69.4	64.3
Temperature	°C	29.15	29.15	29.7	29.6	29.55	29.55	29.3	27.25	26.8	26	29.35	28.25	27.85	28.55
Colour	Hazen	45	22.5	30	25	22.5	25	30	45	20	35	35	25	35	20
Total Dissolved Solids at 105 °C	mg/L	384	205.5	376	246.5	281.5	272	252.5	361.5	227.5	365	203	246.5	603	243.5
Total Suspended Solids	mg/L	7.5	7.5	8	9	9	11	8	10.5	7.5	12.5	10.5	10	9.5	11.5
Ammonia, free	mg/L	0.14	0.12	0.13	0.14	0.11	0.11	0.14	0.1	0.17	0.2	0.1	0.16	0.15	0.14
Dissolved Oxygen (DO)	mg/L	7.1	6.95	7	6.85	6.9	6.85	7.3	7.45	7.55	7.55	7.3	7.35	7.1	7.65
Chemical Oxygen Demand (COD)	mg/L	10.85	6.865	10.5	10.15	8.605	8.4	6.795	11.8	7.72	11	8.6	8.36	9.75	8.945
Biological Oxygen Demand (BOD)	mg/L	4.34	2.745	4.2	3.9	3.44	3.36	2.75	3.8	3.15	4.3	3.45	3.3	3.85	3.55
UV absorbing organic constituents	abs.	0.104	0.065	0.095	0.092	0.076	0.071	0.064	0.114	0.073	0.105	0.0805	0.079	0.091	0.084
UV absorbing organic constituents conc.	mg/L	8.19	5.117	7.535	7.244	6.051	5.629	5.076	8.93	5.769	8.3	6.39	6.1	7.194	6.662
Total Nitrogen (Inorganic, as N)	mg/L	2.055	1.14	2	1.76	1.415	1.28	1.115	2.985	1.345	3.09	1.08	1.605	4.465	1.73

		Mahmodia Canal	Zarkon	End of Khandak Canal	Town of Abou Hommos	Town of Kafr El Dawar	Khorshid intake	Manshia WTP intake	Mahmodia Canal	Zarkon	End of Khandak Canal	Town of Abou Hommos	Town of Kafr El Dawar	Khorshid intake	Manshia WTP intake
		Winter							Spring						
Inorganic Analysis		1	2	3	4	5	6	7	1	2	3	4	5	6	7
Turbidity	NTU	11.75	11	8.9	10.38	11.1	7.61	11.86	14.15	12.7	18.3	14.8	9.1	11.5	9.45
pH value	pH unit	7.6	7.85	7.8	7.625	7.875	7.78	8.435	7.475	7.31	7.48	7.55	7.45	7.6	7.75
Electrical Conductivity at 25 °C	µS/cm	466.5	412	860	466.5	412	860	582	750	410.5	764	858	849	886.5	565
Hardness, total	mg/L	166	152	254	166.2	151.8	253.9	181.8	214	153	234	174	172.5	220	210
Alkalinity, total	mg/L	152.5	144.5	188.5	152.9	144.6	188.3	155.7	165	143.5	181.5	210	230	210	170
Chloride	mg/L	45.5	37.2	65.1	45.5	37.2	65	60.2	43.55	33.75	60.25	61.5	65.25	72	57.65
Phosphate	mg/L	0.215	0.185	0.32	0.214	0.1849	0.317	0.02	0.035	0.055	0.205	0.1	0.07	0.06	0.045
Nitrate	mg/L	8.25	3.75	7.175	9.219	9.7174	24.14	9.703	9.65	4.9	9.6	14.385	15.06	15.79	5.735
Nitrite	mg/L	0.19	0.148	0.37	0.191	0.1485	0.368	0.318	0.29	0.555	0.315	1.15	0.125	0.145	0.1
Silica	mg/L	12.48	5.155	11.775	13.31	6.03	12.39	7.155	6.2	5.86	7.455	11.18	5.06	11.51	5.945
Sulphate	mg/L	35.03	21.45	164	35.01	21.45	163.8	67.12	105.8	24.8	97.59	100.04	122.1	109.9	75.85
Temperature	°C	17	18	17.5	22.85	23.05	22.85	22.9	21.5	21	21.4	22.1	21.7	22.5	23.5
Colour	Hazen	45	55	40	40	35	40	25	37.5	20	45	25	15	30	35
Total Dissolved Solids at 105 °C	mg/L	280	247.5	516	714.5	693.5	735.5	349	450	246	458.5	514.5	509.5	506.5	313.5
Total Suspended Solids	mg/L	9.5	6.5	8.5	10.5	8	9.5	9	15.33	9.25	16.85	9.95	7.9	8.9	9.85
Ammonia, free	mg/L	0.1	0.08	0.09	0.21	0.255	0.26	0.345	1.3	1.25	1.45	0.9	0.17	0.155	0.15
Dissolved Oxygen (DO)	mg/L	6.35	7.1	5.75	6.9	6.85	7.15	7.45	7.45	7.6	7.5	7.6	7.85	7.8	7.95
Chemical Oxygen Demand (COD)	mg/L	18	14	27	17.45	33.6	15.6	16.25	14.05	11.5	16.5	14.2	11.85	12	12.5
Biological Oxygen Demand (BOD)	mg/L	3.85	3.905	4	3.75	4.1	3.35	3.365	4.68	3.83	5.5	4.7	3.945	3.995	4.13
UV absorbing organic constituents	abs.	0.116	0.086	0.0965	0.084	0.2461	0.444	0.067	0.13	0.108	0.158	0.1325	0.1005	0.112	0.12
UV absorbing organic constituents conc.	mg/L	10.1	6.23	8.21	6.656	19.518	7.333	5.339	10.54	8.59	12.45	10.795	8.74	8.905	9.555
Total Nitrogen (Inorganic, as N)	mg/L	2.01	0.96	1.815	2.314	2.451	5.781	2.573	3.225	2.305	3.475	4.285	4.86	3.745	1.445

### Bacteriological and biological analysis

Bacteriological samples were collected in sterilized glass bottles following aseptic techniques to maintain sample integrity. Immediate on-site measurements were prioritized for parameters sensitive to environmental equilibrium. All samples were preserved in ice during field transport and subsequently refrigerated at 4 °C in the laboratory until analysis. Microbiological assessment included: Total coliform (TC) and fecal coliform (FC) quantification (CFU/100 mL) and Analysis performed according to^18^ standard methods. Phytoplankton evaluation involved: Collection using a Rüttner water sampler; Sedimentation technique following (APHA 1999) protocols; Microscopic examination using research-grade microscopes; Species identification and enumeration (expressed as units/L). The zooplankton samples were collected by filtering 100 liters of water through a 55 μm mesh plankton net and preserved with 5% neutral formalin in 250 mL polyethylene bottles. Each sample was concentrated to 100 mL and examined under a microscope. For enumeration, three aliquots (1 mL each) were taken from the sample, transferred to a counting chamber, and the number of individuals per species was counted, expressed as individuals per cubic meter^6,8^.

### Statistical analysis

Statistical analysis was performed using Microsoft Office Excel 2010 and PRIMA 5. The correlation matrix created by the IBM SPSS statistics, version 22 software was used to determine the correlations between various characteristics. At the 0.01 (2-tailed) and 95% confidence levels (*p* < 0.05), the correlation coefficients are deemed significant. The data were subjected to Cluster Analysis (CA) using the MVSP (version 3.1) package application. Ward’s approach (z-transformation of the input data, Euclidean distance as similarity measure) was used to perform cluster analysis based on the standardized mean of 16 physicochemical water parameters observed during the four seasons, such as turbidity, pH, EC, TDS, TSS, NH_3_, COD, BOD, hardness, Total Alkalinity, Cl, PO_4_, NO_3_, NO_2_, Si and SO_4_ were used as variables to show the spatial heterogeneity among the stations.Multivariate statistical analysis was conducted using Cluster Analysis (CA) to classify sampling stations based on similarities in water quality characteristics. The analysis was performed using MVSP software (version 3.1), applying Ward’s linkage method with Euclidean distance as a similarity measure after Z-score standardization of the data. Sixteen physicochemical parameters (turbidity, pH, EC, TDS, TSS, NH₃, COD, BOD, hardness, total alkalinity, Cl⁻, PO_4_^3^⁻, NO_3_⁻, NO_2_⁻, Si, and SO_4_^2^⁻) were used as input variables. Cluster dendrograms were constructed for each season to assess spatial heterogeneity and identify potential pollution hotspots along Al-Mahmoudia Canal.

## Results and discussion

The physicochemical analysis of water samples collected from Mahmoudia Canal and its branches during the four seasons of 2023 revealed noticeable seasonal and spatial variations.PH levels were slightly alkaline in both seasons, ranging from 7.2 to 8.6, which is within the acceptable range for freshwater ecosystems. Turbidity values were generally higher during summer (up to 30 NTU) compared to autumn, spring and winter (autumn, ranging between 8.4 and 19 NTU), (spring ranging between 4.7 and 19.4 NTU, winter, ranging between 6.92 and 15) likely due to increased suspended particles and algal growth under higher temperatures. Electrical Conductivity (EC) values were higher in autumn (up to 1681 µS/cm), indicating increased dissolved ions possibly from agricultural runoff during the post-harvest season. In contrast, EC values during summer, winter and spring were relatively lower, especially in upstream stations. These variations suggest the influence of seasonal factors, such as temperature, rainfall, and anthropogenic activities, on water quality^2^.

### Physio-chemical and biological parameters

The inorganic analysis of water quality across seven locations during four seasons (Summer, Autumn, Winter and Spring seasons) revealed notable spatial and seasonal variations in several physical and chemical parameters. These variations reflect the impact of natural factors, such as temperature and hydrology, as well as anthropogenic influences, such as urban discharge, agricultural runoff, and industrial activities between across seven sampling stations. The results indicate notable differences in several chemical and physical indicators, reflecting seasonal influences, potential pollution sources, and hydrological changes (Alprol et al. 2021)^6,8^.

The current results indicate that the ecological status of Al-Mahmoudia Canal is significantly affected by seasonal variations in physicochemical properties, levels of heavy metals, microbiological pollution, and plankton community changes. These findings bolster the increasing agreement that freshwater ecosystems need to be assessed through integrated environmental assessment methods instead of depending on separate water quality indicators. Recent research has highlighted that the interplay between various physicochemical factors has synergistic impacts on aquatic ecosystem functionality and biological communities, underscoring the need for thorough monitoring approaches for sustainable water resource management^19–22^. Similarly, ecological informatics and multi-parameter decision-support systems have recently been suggested as valuable instruments for enhancing water quality evaluation and recognizing contamination threats in intricate freshwater environments^23,24^.

Furthermore, the noted connections between the decline in water quality and shifts in phytoplankton and zooplankton communities suggest that biological populations react delicately to accumulating environmental pressures. This aligns with recent findings showing that both traditional pollutants and new contaminants can change aquatic ecosystem structures, trigger biological stress, and impact ecological stability, even at relatively low concentrations of individual contaminants^23–25^. Thus, combining biological indicators with physicochemical, microbiological, and heavy metal assessments offers a more dependable assessment of ecosystem health and enhances the scientific foundation for ongoing monitoring and sustainable management of freshwater channels like Al-Mahmoudia Canal.

Turbidity levels were significantly higher in summer, particularly at stations 1, 2 (29.15 NTU) and 3 (29.5 NTU), suggesting intense runoff or resuspension of sediments. Turbidity levels were generally higher in spring, especially at stations 3 and 4 (18.3 and 14.8 NTU, respectively), compared to winter values (max 11.86 NTU at station 7). This increase may result from enhanced surface run off; resuspension of sediments, or biological activity^2^. In autumn, turbidity decreased at most stations, indicating clearer water conditions. Total Suspended Solids (TSS) followed a similar trend, with summer values peaking at station 6 (11 mg/L) and station 3 in autumn (12.5 mg/L), suggesting seasonal sediment loading. Total Suspended Solids (TSS) followed a similar pattern, with spring peaks at stations 3 and 4 (up to 16.85 mg/L) compared to winter maxima of 10.5 mg/L. Total Dissolved Solids (TDS) increased at most stations in spring (e.g., station 4: 514.5 mg/L) vs. winter (e.g., station 4: 246.5 mg/L), again indicating higher mineral or pollutant input.

Due to rainfall and runoff, summer conditions were marked by greater turbidity, suspended particles, and moderate nutrient levels. Nitrate, silica, chloride, and UV-absorbing organics all increased in the autumn, especially at station 6, suggesting a greater human effect. A localized pollution hotspot was suggested by Station 6’s continuously increasing pollutant readings. To preserve ecosystem health and water usefulness, regular monitoring and pollution source management are essential, especially for nutrient control and chloride reduction. El-Zeinyet al.^26^ investigated the anthropogenic impacts on the water quality of the Rosetta branch of the Nile, demonstrating significant changes associated with human activities. Increased nutrient loading, turbidity, and suspended particles were seen in the spring, most likely as a result of biological productivity, runoff, and agricultural activity. While nitrogenous pollution was comparatively reduced throughout the winter, organic pollutant absorption was higher. Elevated nutrient and organic levels were often seen at stations 3, 4, 5, and 6, indicating localized pollution hotspots. Maintaining appropriate water quality requirements and tracking nutrient enrichment need ongoing seasonal monitoring. The importance of dissolved oxygen, biochemical oxygen demand, nitrate, and electrical conductivity as key indicators of water quality variation has also been highlighted in recent water quality assessment approaches, confirming their role as important drivers of aquatic ecosystem conditions^19–22^.

The pH values remained within neutral to slightly alkaline ranges in both seasons (7.7–7.95), indicating stable buffering capacity. It is remained within a slightly alkaline range across both seasons, but was marginally lower in spring, especially at station 2 (7.31), suggesting slightly more acidic or neutral conditions during that season. However, Electrical conductivity (EC) values increased significantly in autumn at station 6 (1004.5 µS/cm), implying higher ionic concentration, possibly due to evaporation, reduced dilution, or localized pollution. Electrical conductivity increased at many sites in spring (e.g., station 6 reached 886.5 µS/cm) compared to winter, indicating higher ionic concentrations, possibly due to evaporation, anthropogenic discharges, or agricultural runoff^11,12^.

Total hardness showed a seasonal increase, especially at station 1 (227.5 mg/L) in autumn, while alkalinity remained relatively stable, indicating consistent carbonate buffering. Station 1 and 3 exhibited the highest hardness and alkalinity values, possibly linked to groundwater influence or geological inputs. Both total hardness and alkalinity were elevated in spring, especially at stations 1, 3, 5, and 6, pointing to increased dissolved mineral content. Station 3 showed the highest hardness (234 mg/L) and alkalinity (230 mg/L), possibly due to geological influence or urban wastewater inputs.

### Nutrients: nitrate, nitrite, ammonia, and total nitrogen

Nitrate concentrations were notably higher in autumn, peaking at station 6 (18.9 mg/L), suggesting increased agricultural runoff or wastewater discharges. Nitrate values increased significantly in spring, with peaks at stations 5 and 6 (15.06 and 15.79 mg/L, respectively), indicating possible fertilizer runoff or sewage contamination. But Nitrite showed an unusual spike at station 4 in spring (1.15 mg/L), which could point to incomplete nitrification or recent organic pollution.

Nitrite and ammonia remained low overall, but slight increases in nitrite at stations 1 and 3 (above 0.5 mg/L) in both seasons may indicate ongoing nitrification processes or partial pollution.

Total inorganic nitrogen was also highest at station 6 in autumn (4.465 mg/L), highlighting a potential eutrophication risk. Total Inorganic Nitrogen was considerably higher in spring, reaching up to 4.86 mg/L (station 5) and 5.78 mg/L (station 6 in winter), indicating eutrophication risks.

Ammonia levels rose sharply in spring, peaking at station 1 (1.3 mg/L) and station 3 (1.45 mg/L), further supporting recent organic loading^27^. Previous Egyptian investigations also reported significant fluctuations in nitrate, nitrite, and ammonia levels in response to seasonal inputs and pollution sources, where nitrate reached up to ~18 mg/L and nitrite up to ~1.5 mg/L in impacted sites, suggesting organic loading and nitrification processes^2^.

Phosphate levels were generally low across all stations (< 0.2 mg/L), yet autumn showed slightly elevated values, particularly at station 1 and 3, indicating increased nutrient loading. Silica showed a remarkable increase in autumn, with peaks at stations 1 and 3 (13.7 and 12.32 mg/L), suggesting inputs from weathering or industrial sources. Silica levels varied, with winter peaks at stations 1 and 3 (12.47 and 11.77 mg/L), and spring levels being lower overall, suggesting reduced mineral input or biological uptake.

Chloride concentrations increased sharply at station 6 in autumn (152 mg/L), possibly due to saline intrusion, industrial effluents, or sewage contamination. Chloride concentrations showed little variation, with modest increases in spring at some stations. Sulphate increased in spring, especially at stations 2–6, with values above 100 mg/L, suggesting potential domestic, detergent, or industrial sources. Sulphate levels were highest in summer at stations 5 and 6, reaching 131.05 mg/L, reflecting possible detergent or fertilizer influence.

### Organic load indicators (COD, BOD, UV-absorbance)

Chemical Oxygen Demand (COD) and Biological Oxygen Demand (BOD) were generally moderate, with summer peaks at station 1 (COD: 10.85 mg/L, BOD: 4.34 mg/L). Autumn showed more balanced organic loads, although elevated values at stations 3 and 6 suggest persistent organic pollution. UV-absorbing organic compounds and their concentrations were higher in autumn, particularly at station 1 (0.1135 absorbance, 8.93 mg/L), indicating presence of dissolved organic matter possibly from industrial or municipal discharge.

Chemical Oxygen Demand (COD) and Biological Oxygen Demand (BOD) levels were slightly lower in spring, suggesting better oxygenation or dilution. However, station 3 maintained high BOD (5.5 mg/L), indicating persistent organic matter presence.

UV-absorbing organic compounds peaked in winter at station 5 (absorbance = 0.44), indicating presence of complex organic pollutants, while concentrations remained high in both seasons, especially at stations 1 and 3.

Dissolved Oxygen (DO) levels remained within healthy ranges (6.85–7.65 mg/L) in both seasons, suggesting good oxygenation. Slightly higher DO in autumn might be linked to lower water temperatures and decreased microbial activity. DO values were higher in spring across all stations, peaking at 7.95 mg/L (station 7), compared to 5.75–7.45 mg/L in winter. This suggests better aeration and lower oxygen demand, likely due to lower microbial respiration rates or cooler, more oxygen-rich water^28,29^.

### Heavy metals

The seasonal variations in heavy metal concentrations across seven sampling stations revealed clear fluctuations throughout the year. Overall, most metals exhibited higher concentrations during the autumn and spring seasons, while lower values were generally observed in winter and summer for several metals. Heavy metals concentrations were reduced^16,17^.

Aluminum (Al) levels showed significant increases during autumn and spring, particularly at stations 4 and 7, where values reached 0.248, 0.267mg/L and 0.332, 0.368mg/L, respectively. In contrast, the lowest values were observed in winter, especially at station 7 (0.025 mg/L). These seasonal increases may be attributed to higher runoff and increased sediment resuspension during rainy periods.

Barium (Ba) concentrations peaked during autumn and spring, with the highest level recorded at station 3 in autumn (0.535 mg/L) and a similar pattern at station 3 in spring (0.485 mg/L). The lowest concentration was observed at station 7 in winter (0.0105 mg/L), suggesting a possible dilution effect during colder months or reduced anthropogenic input.

Copper (Cu) exhibited elevated levels in autumn and spring, particularly at stations 2 and 3, with maximum concentrations of 0.221, 021 mg/L and 0.177, 0.159 mg/L, respectively. Conversely, the lowest values occurred in summer and winter, suggesting reduced inputs from surface runoff or industrial activities during dry and cold periods.

Manganese (Mn) showed an increasing trend in spring, with the highest level observed at station 7 (0.13 mg/L), while the lowest concentrations were noted in summer and winter (0.0075 mg/L at station 4). This pattern may be linked to higher microbial activity and organic matter degradation during warmer months, enhancing Mn mobility.

A marked seasonal variation was observed for molybdenum (Mo). It was highest in summer and winter, reaching 0.5465 mg/L at station 5 and lowest in spring (0.010 mg/L at station 5). The decline in spring suggests possible biological uptake or reduced inputs during that season.

Vanadium (V) concentrations were relatively low in summer and winter, with values between 0.004 and 0.0175 mg/L, but increased sharply in spring, especially at station 6 (0.4575 mg/L). This rise may be associated with enhanced erosion or increased industrial discharge in the region during this season.

Zinc (Zn) levels showed a clear decline from summer to spring, with the highest concentrations in summer (up to 0.064, 0.066 mg/L at station 4), and disappear in spring at station 1, 2). This reduction could be due to biological uptake or sedimentation processes becoming more active during the warmer seasons (Table 2).

**Table 2:** Seasonal variations of heavy metals parameters (water quality) recorded at all sites at Al-Mahmoudia Canal

			Mahmodia Canal	Zarkon	End of Khandak Canal	Town of Abou Hommos	Town of Kafr El Dawar	Khorshid intake	Manshia WTP intake	Mahmodia Canal	Zarkon	End of Khandak Canal	Town of Abou Hommos	Town of Kafr El Dawar	Khorshid intake	Manshia WTP intake
			Summer							Autumn						
Heavy Metals Analysis			1	2	3	4	5	6	7	1	2	3	4	5	6	7
Aluminium	Al	mg/L	0.09	0.0415	0.1	0.051	0.04	0.04	0.03	0.14	0.16	0.151	0.248	0.1	0.17	0.332
Barium	Ba	mg/L	0.07	0.069	0.11	0.042	0.06	0.06	0.11	0.51	0.5	0.535	0.415	0.41	0.43	0.405
Copper	Cu	mg/L	0.01	0.02	0.03	0.052	0.03	0.02	0.01	0.16	0.22	0.177	0.055	0.16	0.1	0.055
Manganese	Mn	mg/L	0.03	0.025	0.04	0.008	0.01	0.02	0.01	0.02	0.06	0.0235	0.097	0.04	0.1	0.049
Molybdnum	Mo	mg/L	0.38	0.43	0.28	0.353	0.54	0.41	0.38	0.11	0.31	0.255	0.35	0.16	0.41	0.064
Vanadium	V	mg/L	0	0.0045	0.01	0.005	0.01	0.01	0.01	0	0	0.0067	0.01	0.01	0.01	0.011
Zinc	Zn	mg/L	0.05	0.03	0.04	0.064	0.06	0.04	0.06	0.01	0.01	0.015	0.024	0.01	0.01	0.01

			Winter							Spring						
Heavy Metals Analysis			1	2	3	4	5	6	7	1	2	3	4	5	6	7
Aluminium	Al	mg/L	0.04	0.0315	0.07	0.031	0.03	0.03	0.03	0.13	0.14	0.138	0.276	0.11	0.19	0.368
Barium	Ba	mg/L	0.05	0.04	0.1	0.067	0.06	0.06	0.01	0.47	0.46	0.485	0.46	0.45	0.47	0.425
Copper	Cu	mg/L	0.02	0.02	0.04	0.035	0.03	0.02	0.02	0.14	0.21	0.159	0.052	0.15	0.09	0.052
Manganese	Mn	mg/L	0.04	0.051	0.05	0.008	0.01	0.02	0.01	0.03	0.03	0.0435	0.105	0.04	0.08	0.13
Molybdnum	Mo	mg/L	0.41	0.43	0.28	0.351	0.55	0.39	0.34	0.02	0.06	0.02	0.107	0.01	0.02	0.055
Vanadium	V	mg/L	0.02	0.0045	0.01	0.005	0.01	0.01	0.01	0.1	0.27	0.23	0.355	0.17	0.46	0.07
Zinc	Zn	mg/L	0.04	0.025	0.04	0.066	0.05	0.04	0.06	0	0	0.0065	0.017	0.01	0.01	0.012

### The bacteriological measurement

The bacteriological analysis of water samples across seven stations during different seasons (summer, autumn, winter, and spring) revealed significant spatial and temporal variations in microbial contamination. Three key microbiological indicators were evaluated: Heterotrophic Plate Count (HPC), Total Coliforms, and Fecal Coliforms.

Heterotrophic Plate Count (HPC) at 35 °C after 48 hours values were highest during summer and autumn, indicating favorable conditions for bacterial growth such as higher temperatures and possibly increased organic matter. The maximum count was recorded at station 6 in summer (80,100 CFU/mL) and station 1 in autumn (49,050 CFU/mL), suggesting higher microbial activity and possible anthropogenic inputs during these seasons. In contrast, the lowest values were found during spring, particularly at station 7 (105 CFU/mL), indicating reduced bacterial growth likely due to lower nutrient availability or improved water conditions.

Total coliforms showed wide variation across seasons and stations. The highest concentrations were observed in summer and autumn, particularly at stations 1 and 2, with values reaching 54,200 CFU/100 mL and 62,500 CFU/100 mL, respectively. These results suggest contamination likely related to surface runoff or sewage influence. In contrast, the lowest values were recorded in spring and summer, at station 7 (750, 800 cfu/100 mL), reflecting relatively improved water quality or reduced pollution sources.

Fecal coliform levels, which indicate recent fecal contamination and pose serious public health risks, were notably elevated during spring and autumn. Station 1 in spring recorded the highest level (7170 CFU/100 mL), followed by station 3 (7095 CFU/100 mL), suggesting potential sewage discharge or agricultural runoff. The lowest values were observed in autumn at station 7 (45 CFU/100 mL), potentially due to dilution effects or reduced input of fresh fecal matter (Table 3).

**Table 3:** Seasonal variations of biological analysis of different stations in the study area

		1	2	3	4	5	6	7	1	2	3	4	5	6	7
Bacteriological		Summer							Autumn						
Heterotrophic plate count at 35 °C after 48 h	CFU/1 mL	7550	3200	3150	57000	76500	80100	3600	49050	3550	32050	2800	4300	20500	4000
Total coliform	CFU/100 mL	54200	4200	33500	37500	47000	22550	800	62500	2300	33900	4100	9000	9500	3350
Fecal coliform		3710	75	2235	1010	1675	835	110	4975	90	5510	80	225	350	45

		Winter							Spring						
Heterotrophic plate count at 35 °C after 48 h	CFU/1 mL	10050	2550	2190	20700	24000	9250	5400	30800	1065	5400	725	685	640	105
Total coliform	CFU/100 mL	13250	2350	1650	1550	61000	3850	4200	6250	2500	60350	9750	12200	9550	750
Fecal coliform		2360	255	140	510	3450	620	635	7170	160	7095	2010	1210	2905	60

High HPC and coliform counts in the summer and autumn indicate increased microbial activity and contamination, most likely as a result of warm weather and pollution. With a few outliers, such as high coliforms at stations 6, 5 microbial loads are generally lower throughout winter and spring. Despite generally improved water quality, mixed trends in the spring-low HPC but high fecal coliforms at several stations-indicate potential sources of contamination. These findings emphasize the necessity for routine monitoring and efficient wastewater treatment, particularly during the warmer months when microbiological contamination is higher, by highlighting the seasonal variability and localized pollution patterns.

Phytoplankton abundance and composition were assessed across four seasons (summer, autumn, winter, and spring) at seven sampling stations. The main groups analyzed included blue-green algae (Cyanobacteria), green algae, and diatoms, with data indicating significant seasonal and spatial variations in total phytoplankton abundance and group dominance.

The total phytoplankton count ranged markedly between stations and seasons. The highest abundance was observed in spring at station 4 (2871.5 Unit/mL), followed closely by winter values at station 5 (2507 Unit/mL). These peaks suggest favorable environmental conditions, such as nutrient enrichment or temperature changes that stimulate growth. Conversely, the lowest total counts were recorded during summer, particularly at station 4 (599 Unit/mL), possibly due to high temperatures or grazing pressure limiting phytoplankton development.

Blue-Green Algae (Cyanobacteria) were dominant in summer and autumn, particularly at station 2 in summer (1084.5 Unit/mL) and station 1 in autumn (1153.5 Unit/mL). These values suggest that eutrophic conditions and high temperatures during these seasons promote blooms. However, their abundance dropped significantly in winter and spring, with the lowest values recorded at station 6 in winter (39 Unit/mL) and station 7 in autumn (47.5 Unit/mL), indicating sensitivity to cooler temperatures or competition with other algal groups.

Green algae showed more even distribution across seasons, with peaks in spring and winter, especially at station 5 in winter (854.5 Unit/mL) and station 3 in spring (775.5 Unit/mL). These groups appear to thrive under moderate conditions and may be more resilient to environmental fluctuations compared to cyanobacteria. Summer levels were generally lower but still present, suggesting continuous growth potential.

Diatoms were consistently dominant in winter and spring, with exceptional abundance in winter at station 1 (1436.5 Unit/mL) and spring at station 3 (1354 Unit/mL). Their dominance in cooler seasons is consistent with their preference for lower temperatures, high turbulence, and increased nutrient availability. In contrast, summer diatom counts were lower, particularly at station 1 (288 Unit/mL), reflecting seasonal shifts in environmental parameters (Table 4).

**Table 4:** Seasonal variations of phytoplankton community of different stations in the study area

		1	2	3	4	5	6	7	1	2	3	4	5	6	7
Phytoplankton		Summer							Autumn						
Total count	Unit/mL	1191	1557.5	1380	599	714	1082	743	2137	883.5	1666	1040.5	1108	806.5	959.5
Blue-Green algae		618	1084.5	609	96	128	112	199	1154	183.5	727.5	134.5	99	122.5	47.5
Green algae		132	68	105	134.5	128	95.5	99	193	189	236	234	297.5	303.5	241.5
Diatoms		288	360.5	593	322.5	420	783	404	359	400	452.5	464.5	529.5	334	534.5

		Winter							Spring						
Total count	Unit/mL	2078	1302	1203	1292	2507	1104	1049	1390	707.5	2371.5	2871.5	551.5	1367	1319.5
Blue-Green algae		280	236.5	109.5	363.5	479.5	39	132	58	122.5	161.5	650.5	112	258	157
Green algae		289	333.5	277	297	854.5	340.5	187.5	610	133	775.5	736.5	106	407.5	454
Diatoms		1437	607.5	699	553.5	888.5	656.5	680.5	693.5	413.5	1354	1001	304.5	631.5	555

In summer, cyanobacteria predominate at certain locations and total phytoplankton counts are lower. Moderate to high cyanobacteria presence in autumn; green algae and diatoms start increasing. Winter, high diatom dominance, with noticeable increases in total counts and green algae. Spring, weak phytoplankton abundance, with strong contributions from both green algae and diatoms, reflecting highly productive conditions.

These observations highlight the dynamic structure of phytoplankton communities, influenced by seasonal changes in temperature, nutrient availability, and water column stability. The dominance of diatoms in winter/spring and cyanobacteria in summer/autumn follows expected ecological succession patterns in temperate aquatic systems.

### Zooplankton community

Patterns of Species Abundance and Richness in summer: During the summer, 34 zooplankton taxa were recorded across seven stations, with total counts ranging from 13,000 to 82,000 individuals. Station 3 had the highest abundance (82,000 in summer), while station 7 had the lowest (13,000 in winter). These high summer abundances align with studies noting that zooplankton-especially rotifers and cladocerans-tend to peak in warmer and more productive seasons.

Predominant Taxonomic Classes: Rotifers (e.g., *Brachionus angularis*, Keratella tropica, *Synchaetaokai*) collectively formed the bulk of the community, reflecting the global trend of rotifer dominance in eutrophic freshwater systems. Copepods (e.g., *Cyclops vernalis*, *Nauplius larvae*) appeared in high numbers at select stations (notably station 6), suggesting localized blooms. Cladocerans, including *Bosmina longirostris* and *Moina micrura*, were widespread but less abundant-typical of summer communities where rotifers often outcompete them under high nutrient loads.

Spatial Variability Amongst Stations: Station 3 exhibited the highest abundance of certain taxa like Metamorphosis (12,000) and Nauplius larvae (19,000), pointing to favorable local conditions possibly nutrient hotspots or reduced predation pressure. Station 6 also saw elevated numbers of *Synchaetaokai* (13,000) and *Cyclops vernalis* (17,000), indicating microhabitat variability, a common feature in heterogeneous lake systems. Stations 1, 2, and 5 were moderate in overall abundance, while stations 4 and 7 were comparatively lower.

Seasonal Factors and Their Effects on the Environment**:** The dominance of rotifers in summer corresponds to the literature, which highlights their rapid reproduction and ability to exploit high phytoplankton levels and organic matter. Copepod and cladocerans populations, though lower, play crucial ecological roles especially copepod larvae for carbon and nutrient cycling. Spatial variation in species abundance suggests differences in local water quality, nutrient availability, or predation intensity, consistent with ecological filtering concepts.

Seasonal Succession: Similar studies note that rotifer densities peak in summer or autumn, while cladocerans and copepods may dominate in other seasons—confirming our summer pattern. Eutrophic Signals: Dominance of Brachionus and Keratella-common in eutrophic lakes-reinforces the interpretation of high nutrient conditions in the canal system. Environmental Indicators: Variable species distributions among stations align with findings that zooplankton taxa respond to both seasonal changes and local environmental factors such as trophic status, flow regimes, and habitat structure (Table 5).

**Table 5:** Seasonal variations of zooplankton community of different stations in the study area

Zooplankton	Summer							Winter						
Species (Sp)	1	2	3	4	5	6	7	1	2	3	4	5	6	7
Rotifera														
Keratellacochlearis	0	5000	0	3000	0	8000	0	0	2000	0	4000	0	0	0
Keratellatropica	2000	0	8000	0	3000	7000	0	0	3000	0	6000	0	3000	5000
Keratellaquadrata	0	1000	0	5000	0	0	0	2000	0	0	0	0	0	0
Brachionuscaudatus	0	2000	0	1000	0	0	4000	0	0	0	0	0	0	0
Brachionusbadapesteninsis	4000	0	0	0	0	0	0	0	0	0	0	0	0	0
Brachionuscalyciflorus	0	7000	0	3000	0	5000	0	0	0	0	0	1000	0	0
Brachionusangularis	0	14000	8000	0	0	0	0	0	0	3000	0	0	0	0
Brachionusurceolaris	0	3000	1000	0	16000	0	0	0	0	7000	0	0	0	0
Brachionusquadridentatus	8000	0	0	0	0	0	14000	0	0	0	0	0	0	0
Metamorphosis	6000	0	12000	0	0	2000	0	4000	0	7000	0	5000	2000	0
Polyarthravulgaris	2000	0	0	0	0	0	0	0	0	0	0	0	0	0
Syncheataokai	0	4000	0	0	0	13000	0	0	0	0	0	7000	0	0
Synchaetaoblonga	5000	0	1000	0	0	0	11000	9000	0	0	0	0	0	3000
Filinialonogiseta	0	0	0	0	0	0	0	0	0	0	0	0	0	0
Asplanchnapriodonta	7000	0	0	13000	0	1000	0	0	7000	0	0	1000	0	4000
Horaellabrehmi	0	0	5000	0	17000	0	0	0	11000	0	0	0	0	0
Proalesdecipiens	0	0	1000	0	0	1000	6000	0	0	0	0	0	6000	0
Copepod														
Halycyclopsmagnisips	10000	2000	0	0	0	0	0	0	4000	0	8000	0	0	0
Naupliuslarva	0	0	19000	0	1000	0	0	0	0	0	0	3000	0	0
Cyclopsvernalis	2000	0	0	0	0	17000	0	0	0	0	0	0	0	0
Cladocera														
Euterpinaaquifrons	1000	1000	13000	0	0	0	1000	8000	0	0	0	0	0	1000
Bosminalongirostris	0	5000	0	0	0	0	0	0	0	0	0	0	0	0
Alonellaexcisa	4000	0	0	7000	3000	13000	0	0	5000	0	0	0	7000	0
Moinamicrura	0	1000	0	0	0	0	12000	0	0	0	0	0	0	0
Bosminalongirostris	0	0	9000	0	0	0	0	0	0	0	0	0	0	0
Ostracoda														
Cycrediapunitellata	5000	0	0	0	15000	0	0	0	0	0	13000	0	0	0
Thycrediapunitullata	0	0	3000	0	0	0	0	0	0	9000	0	0	0	0
Mollusca														
Veligaroflamellibranchiata	5000	0	0	2000	0	7000	0	7000	0	0	1000	0	1000	0
Nematode														
Freelivingnematoda	0	3000	0	0	1000	0	5000	8000	0	0	0	0	11000	0
Polycheatasp	2000	0	0	0	0	0	0	0	0	0	0	0	0	0
Chordate														
FishEggs	3000	0	0	0	7000	0	0	0	0	0	0	0	0	0
Protozoa														
Glopigerinainflata	0	4000	0	0	0	5000	0	0	0	0	0	0	4000	0
Centropixisaculata	7000	0	2000	6000	0	0	4000	4000	0	5000	0	2000	0	0
Total	73000	52000	82000	40000	63000	79000	57000	42000	32000	31000	32000	19000	34000	13000

Zooplankton	Spring								Autumn					
Species[Sp]	1	2	3	4	5	6	7	1	2	3	4	5	6	7
Rotifera														
Keratellacochlearis	2000	0	0	0	3500	0	0	3000	0	1000	0	4000	0	0
Keratellatropica	0	3000	0	2000	0	2500	4500	2000	0	4500	1500	0	500	4500
Keratellaquadrata	2000	0	0	500	0	0	0	1500	0	0	3000	0	0	0
Metamorphosis	0	1500	1000	2000	0	0	0	2000	0	0	500	0	0	0
Brachionuscaudatus	0	0	0	0	4500	0	1500	0	0	0	0	0	0	2000
Brachionusbadaqstenisinis	500	0	0	0	0	0	0	0	0	0	0	0	0	0
Brachionuscalyciflorus	0	0	2000	0	500	0	0	0	4000	3500	0	1000	0	0
Brachionusangularis	0	2000	3500	0	0	0	0	0	3000	0	2500	0	0	0
Brachionusurcorialis	0	0	0	0	0	0	0	0	0	0	0	0	0	0
Brachionusurcorialis	0	0	0	0	0	0	0	0	1500	0	3000	0	0	0
Brachionusquadridentatus	0	0	500	0	0	0	0	4500	0	5000	0	0	0	1000
Polyarthravulgaris	4000	0	0	0	0	0	0	0	0	0	0	0	0	0
Synchetaoikai	0	0	4000	0	2500	0	0	0	2000	0	0	2500	0	0
Filinialongiseta	0	1000	0	0	0	0	0	0	0	0	500	0	0	0
Synchetaoblonga	0	0	0	4500	0	0	1000	0	0	0	0	0	0	2500
Asplanckiaprilociordata	1500	1500	0	6500	0	3500	0	1000	0	0	0	3500	0	1500
Horaxellabrehmi	0	0	0	0	0	0	2000	0	0	0	0	1000	0	0
Proalesdecipiens	0	0	1500	0	0	4000	0	0	2000	0	0	0	2000	0
Copepod														
Halycyclopsmagnisops	2500	0	0	0	0	0	0	0	0	0	0	0	0	0
Naupliuslarva	0	0	0	0	3000	0	3500	0	0	0	0	500	0	0
Cyclopsvernalis	0	0	0	0	0	3000	0	5000	0	3500	0	0	4000	0
Cladoera														
Euterpiniaaquitifrons	0	2000	0	0	0	0	0	0	500	0	1500	0	0	6000
Bosminalongirostris	0	0	0	5500	0	0	1000	0	0	0	0	0	0	0
Alonellaexcisa	6000	0	1000	0	0	500	0	0	0	0	500	1500	4000	0
Moinamicrura	0	2500	0	0	1500	0	0	2000	0	3000	0	0	0	1000
Bosminalongirostris	0	0	0	0	0	0	0	0	0	0	1000	0	0	0
Ostracoda														
Excreliapuncellata	0	0	0	0	0	0	0	0	0	0	0	1000	0	0
Thycreliapuncellata	0	0	5000	0	0	0	5500	500	0	0	0	0	0	0
Mollusca														
Veiligaroflamelliibranchiata	3000	0	0	2000	0	1500	0	2000	0	500	0	0	1000	0
Nematode														
Freelivingnematoda	0	0	0	3500	0	0	0	0	500	0	1500	3000	0	1500
Polychatasp	0	0	0	0	0	0	0	0	0	0	0	0	0	0
chordate														
FishEggs	0	4000	0	0	0	0	0	0	3500	0	0	0	0	0
Protozoa														
Gloepigenainflata	0	0	0	0	4500	0	0	0	0	0	0	0	2000	0
Coriostrovisacutata	1500	0	1500	3500	0	0	0	0	1500	2500	3000	0	3000	0
Total	23000	17500	20000	30000	20000	15000	19000	23500	18500	23500	18500	18000	16500	20000

Summer zooplankton communities are dominated by rotifers, especially Brachionus and Keratella, indicating warm, nutrient-rich conditions. Copepod and cladocerans presence suggests functional diversity and ecological complexity. Spatial heterogeneity across stations underscores the need for targeted environmental monitoring and management-especially in nutrient hotspots like stations 3 and 6. For future studies, consider seasonal sampling, nutrient-water quality correlations, and diversity indexing to track ecosystem health. The total abundance of zooplankton showed clear seasonal variation. The summer season recorded the highest total abundance, particularly at station 3 (82,000 individuals), followed by spring and autumn (each up to 23,500 individuals), while winter showed the lowest abundance, with only 13,000 individuals at station 7. Several species, such as Metamorphosis, *Keratella tropica*, and *Brachionus angularis*, were notably dominant during summer, reflecting favorable environmental conditions. In contrast, winter samples exhibited lower richness and density, with some taxa like *Polyarthra vulgaris* and *Filinialongiseta* being rare or absent. Zooplankton composition and abundance varied significantly across stations within the same season. For example, station 3 in summer and station 5 in both spring and autumn recorded high species richness and densities, while others remained low. This variability is likely influenced by localized physicochemical conditions such as nutrient availability, temperature, and water movement. Seasonal patterns were evident, with distinct communities observed in each season, indicating a strong influence of temporal environmental shifts on community structure.

Keratella tropica, *Brachionus angularis*, and *Asplanchna priodonta* were dominant species in summer and spring, reflecting their preference for warmer, nutrient-rich waters. *Cyclops vernalis* and *Nauplius larvae* were more frequent during winter and spring, suggesting their life cycles are synchronized with colder water conditions. Some copepods, such as *Halycyclops magniceps*, were abundant in winter and spring, indicating their adaptability to varying environmental parameters.

Environmental Influence on Zooplankton Composition, the observed seasonal shifts in zooplankton assemblages align with previous findings that temperature, dissolved oxygen, and nutrient concentrations are key drivers of plankton dynamics^30,31^. Elevated temperatures during summer likely enhance metabolic and reproductive rates of rotifers, leading to higher abundance of taxa like Brachionus and Keratella. Conversely, lower temperatures in winter result in reduced zooplankton activity and diversity, as noted in many freshwater systems^32^.

Certain rotifers such as *Brachionus calyciflorus* and *Keratella cochlearis* are considered indicators of eutrophic conditions, due to their tolerance to fluctuating nutrient levels^33^. In contrast, taxa like Polyarthra and Synchaeta are typically associated with mesotrophic to oligotrophic environments^34^, suggesting variable water quality across sites and seasons. The presence and dominance of specific taxa can thus serve as bioindicators, reflecting the ecological health and trophic status of the studied aquatic environment. Seasonal and spatial variation in zooplankton communities reflects the dynamic nature of the aquatic ecosystem. Summer supports the highest species richness and density due to elevated temperature and productivity, while winter shows reduced diversity due to environmental stress. These findings highlight the ecological significance of zooplankton as sensitive indicators of environmental changes, and reinforce the value of integrating biological assessments with physicochemical monitoring.

Analysis of zooplankton communities showed seasonal differences in species richness and diversity indices: The analysis of zooplankton community structure across seven stations revealed marked spatial variation in both species’ richness and abundance, as well as diversity indices. The metrics evaluated included total species number, total individual count, Simpson’s dominance index (D), species richness, evenness (J), Fisher’s alpha, and Shannon-Wiener diversity index (H) (Table 6).

**Table 6:** The diversity indices between the selected sites in El-Mahmoudia canal during this study

Stations	Total species	Total individuals	D richness	J evenness	Fisher	H
1	11	40,375.00	0.90	0.94	1.00	2.16
2	9	30,000.00	0.78	0.92	0.87	1.99
3	9	39,125.00	0.72	0.92	0.80	1.90
4	8	30,125.00	0.71	0.90	0.79	1.87
5	8	30,000.00	0.64	0.88	0.72	1.77
6	8	36,125.00	0.65	0.91	0.73	1.83
7	7	27,250.00	0.57	0.91	0.64	1.70

Station 1 recorded the highest species richness (11 species) and total individual count (40,375 individuals), indicating favorable environmental conditions and possibly higher habitat heterogeneity or food availability. In contrast, station 7 showed the lowest richness (7 species) and lowest individual count (27,250), suggesting less favorable conditions, such as limited resources, habitat degradation, or pollution stress.

Indices of diversity, Simpson’s Dominance Index (D) values ranged from 0.57 (station 7) to 0.90 (station 1). Higher values indicate greater dominance of certain species, while lower values suggest more even distribution. Species richness index values were highest at station 1 (0.94), followed closely by station 2 (0.92), showing a relatively balanced community with a variety of species. Evenness (J) remained consistently high across stations (0.88–0.94), indicating that species were relatively equally distributed in most sites, although slightly lower evenness was noted at stations 4 and 5.

Fisher’s alpha, another measure of species’ diversity, ranged from 1.00 (station 1) to 0.64 (station 7). This again confirms station 1 as the most diverse and station 7 the least. Shannon-Wiener Index (H), a widely used diversity metric, followed the same pattern, peaking at station 1 (2.16) and decreasing to 1.70 at station 7.

There was a clear gradient of decreasing diversity and richness from station 1 toward station 7. This may reflect increasing environmental stress or anthropogenic pressure in downstream or offshore areas. Stations 2 and 3 also maintained relatively high values, indicating moderately healthy zooplankton communities, whereas stations 5–7 showed signs of reduced diversity and abundance, possibly due to eutrophication, low oxygen levels, or contamination.

The zooplankton data reflect a healthy and diverse community at station 1, with progressively reduced diversity and species richness toward station 7. The combined use of diversity indices (Simpson, Shannon, Fisher, evenness) provides a comprehensive understanding of community structure and ecological status. These findings highlight the importance of regular biological monitoring to detect early signs of ecological imbalance and to guide management and conservation efforts. The higher species richness and diversity in summer may be attributed to favorable temperature and nutrient availability supporting zooplankton growth and reproduction. Autumn samples showed a decline in both individual numbers and species count, potentially due to changes in water quality parameters or predation pressure. The evenness values in both seasons indicate a relatively balanced community structure, where no single species overwhelmingly dominates.

Seasonal and spatial patterns in Egyptian freshwater systems have demonstrated significant associations between zooplankton diversity and physico-chemical conditions. For instance, studies in the lakes revealed seasonal variations in zooplankton assemblages linked to environmental parameters^35^. Assessments of zooplankton community structure in fresh water showed that species abundance and diversity corresponded with water quality gradients^2^. Research on rotifer communities at El-Mahmoudia Canal further supports the influence of local ecological conditions on zooplankton composition^2^. Moreover, work on Mariout Lake demonstrated how ecological interventions and water quality shifts can substantially alter zooplankton diversity^11,12^.

The ecological health of Mahmoudia Canal is influenced by both natural seasonal dynamics and human activities such as agriculture and urban discharge. Monitoring zooplankton diversity alongside physicochemical parameters provides valuable insight into ecosystem status and potential stressors.

### Cluster analysis (site grouping and geographic similarity)

**Fig. 2:**
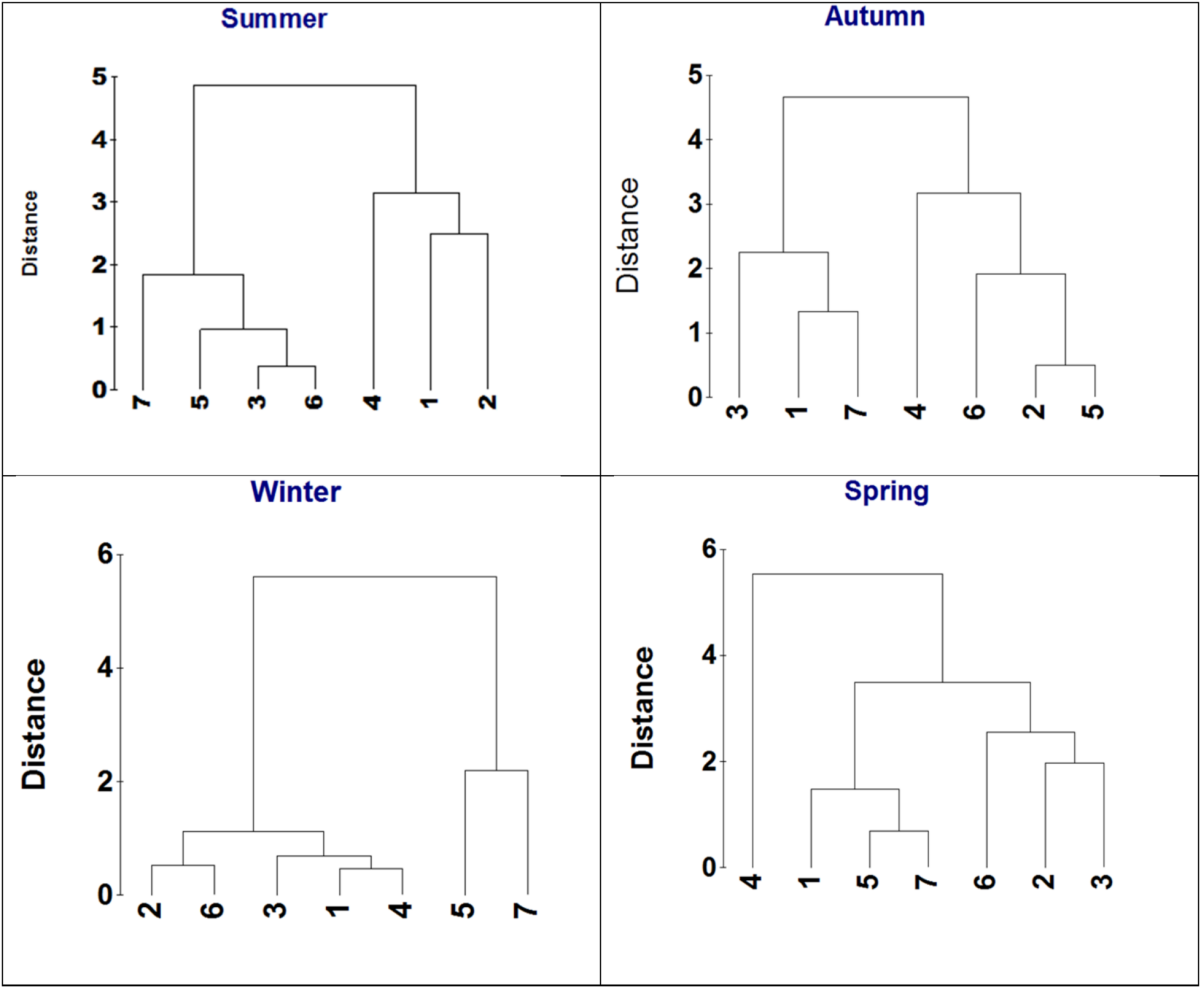
Dendrogram viewing the relationship amongst sampling locations of four seasons in Mahmoudia canal.

Cluster analysis is an unsupervised multivariate statistical technique used to classify the objects into clusters based on their similarity and it is one of the most widely used multivariate statistical technique to evaluate the surface water quality and it is typically illustrated by a dendrogram. Data were then amalgamated into dendrogram plots and the similarity coefficients of all the investigated stations on the Mahmoudia canals base on their water quality similarities are given in Fig. 2. According to the results of CA, two major clusters were documented during summer season. The first cluster were stations 7, 5, 3, 6. The first sub cluster were station 5, 3, 6 and the second sub-cluster was between stations 3 and 6, highest similarity. The second cluster included three stations 4, 1, 2 but sub-cluster was between stations 1 and 2, highest similarity.

During the autumn, the data were found to be similar to that obtained in the summer with slight differences in the arrangement of stations were two major clusters. The first cluster were stations 3, 1, 7. The first sub cluster were station1, 7 highest similarities. The second cluster included four stations 4, 6, 2, 5 but sub-cluster was between stations 1 and 2, highest similarity and the second sub-cluster were between stations 2 and 5 highest similarity.

During the winter and spring found three major clusters were documented. In winter, the first cluster was between stations 2, 6. The second cluster was between stations 3, 1, 4. The third cluster was between stations 5, 7. While in spring, the first cluster was between stations 1 and other stations. The second cluster was between stations 1, 5, 7. The third cluster was between stations 6, 2, 3, therefore the differences between the groups indicate the differences in the sources of pollution. The contribution of metal-related variables in multivariate analyses is important for identifying pollution gradients and understanding the combined effects of physicochemical factors on aquatic ecosystem conditions. Recent data-driven approaches have similarly emphasized the importance of interpreting variable contributions and interactions to assess heavy metal pollution patterns and ecological risks^19–22^.

## Multivariate statistical analysis

### Seasonal variation of water quality based on PCA

**Fig. 3:**
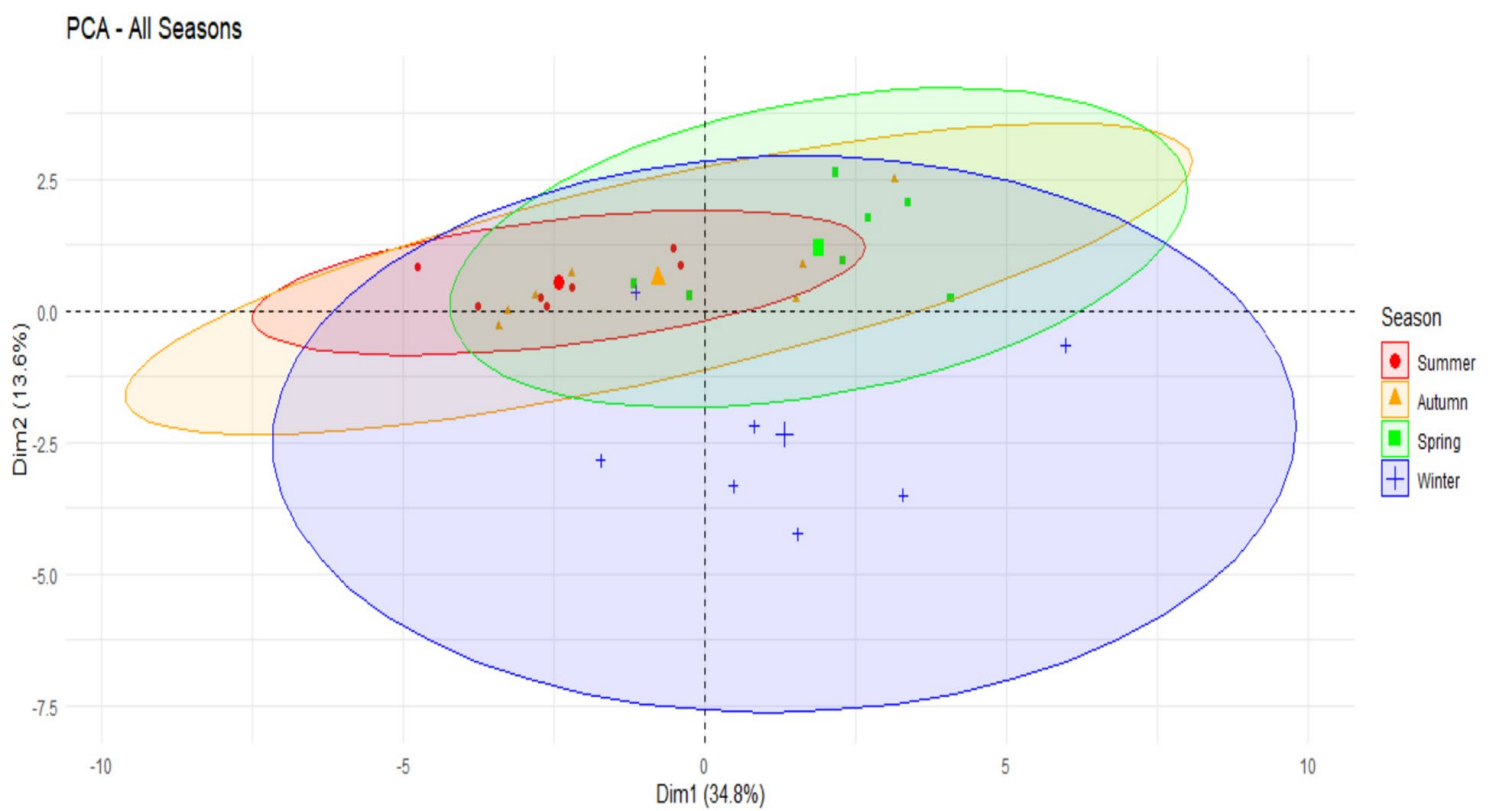
Principal Component Analysis (PCA) of water quality parameters across different seasons.

Principal Component Analysis (PCA) was applied to explore the overall patterns of seasonal variability and to identify the main environmental gradients controlling water quality across all sampling stations (Fig. 3). The first two principal components (Dim_1_ and Dim_2_) together accounted for 48.4% of the total variance, with Dim_1_ explaining 34.8% and Dim_2_ explaining 13.6% of the variability in the dataset. The PCA ordination clearly separated the sampling stations according to seasons, indicating strong seasonal control over water quality characteristics. Winter samples were distinctly separated along the negative side of Dim_2_, forming a well-defined cluster with minimal overlap with other seasons. This separation reflects relatively stable physicochemical conditions during winter, likely associated with lower temperatures, reduced biological activity, and enhanced dilution. In contrast, summer and autumn samples showed partial overlap but were generally positioned on the negative side of Dim_1_, suggesting similar environmental conditions influenced by increased nutrient loading, turbidity, and organic matter during warmer periods. Such clustering indicates intensified anthropogenic pressure, particularly from agricultural runoff and domestic wastewater inputs, which are known to peak during these seasons. Similar seasonal grouping patterns have been documented in multivariate analyses of Egyptian freshwater systems^16,17^. Spring samples occupied a transitional position between winter and summer clusters, mainly distributed along the positive side of Dim_1_ and Dim_2_. This pattern reflects seasonal recovery and mixed environmental conditions, where increased nutrient availability supports biological productivity, while moderate temperatures and hydrological conditions reduce extreme pollution effects. Multivariate statistical techniques such as Pearson correlation analysis and principal component analysis (PCA) are widely used to explore the relationships between heavy metals and physicochemical parameters and to identify pollution sources in aquatic environments^36^. Recent statistical and data-driven approaches have emphasized the importance of evaluating the contribution of individual water quality variables to overall ecosystem variation, where parameters such as electrical conductivity and total dissolved solids are considered, key indicators reflecting dissolved material inputs and changes in aquatic environmental conditions^19–22^.

### Identification of environmental gradients using PCA biplot for all seasons

**Fig. 4:**
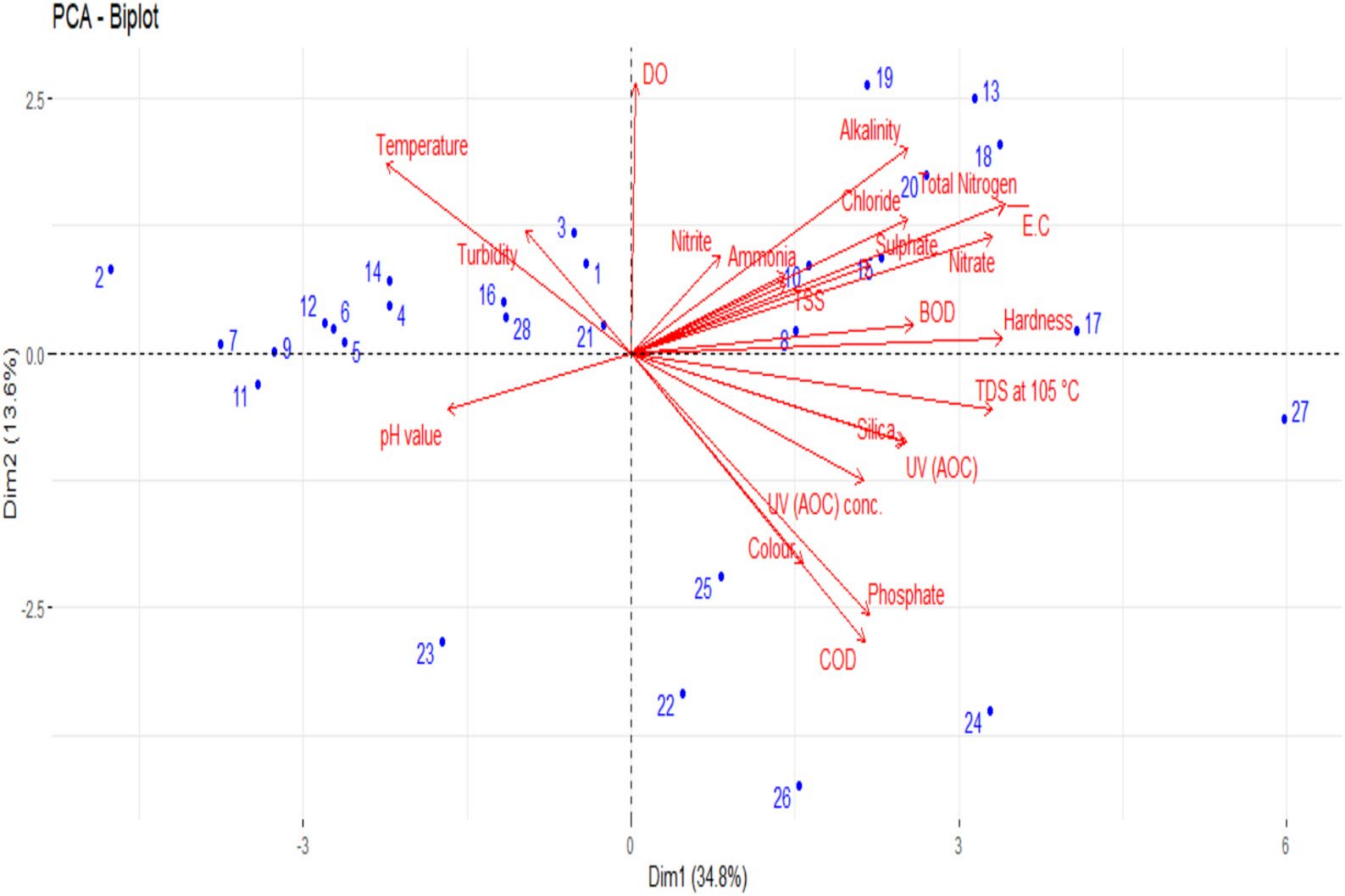
PCA biplot showing the relationships between water quality parameters and sampling sites.

The PCA biplot illustrates the relationships between water quality parameters and sampling sites (Fig. 4), as well as the contribution of each variable to the overall variation of the dataset. The first two principal components (Dim1 and Dim2) explained 48.4% of the total variance, with Dim1 accounting for 34.8% and Dim2 accounting for 13.6%, indicating that these components represent the main environmental gradients affecting water quality variation in Al-Mahmoudia Canal. Dim1 was mainly associated with a pollution and nutrient enrichment gradient, showing strong positive loadings for electrical conductivity (EC), total dissolved solids (TDS), alkalinity, hardness, total nitrogen, nitrate, sulfate, BOD, and COD. The association of these variables suggests the influence of nutrient inputs, organic matter accumulation, and anthropogenic activities such as agricultural runoff and wastewater discharge. Sites positioned on the positive side of Dim1 were characterized by higher dissolved loads and organic enrichment. High COD values indicate increased organic matter inputs from domestic and industrial sources, reflecting the influence of wastewater discharge on water quality degradation^36^. In contrast, variables such as pH, turbidity, and temperature showed higher contributions toward the opposite direction of Dim1, reflecting different physicochemical conditions that may be related to natural variation and dilution effects. Dim2 represented another environmental gradient related to oxygen availability and nutrient dynamics, where dissolved oxygen (DO) showed an opposite pattern to COD, phosphate, silica, and UV absorbance (AOC). This inverse relationship suggests that increased organic and nutrient loading may enhance oxygen consumption and influence the ecological status of the canal. The orientation and angles between vectors indicated relationships among variables; closely aligned vectors represented positive associations, while opposite directions indicated inverse relationships. For example, the strong association between EC, TDS, and nitrogen compounds reflects their common contribution from dissolved inputs, whereas the opposite position of DO against organic pollution indicators indicates the effect of oxygen demand on water quality. The distribution of sampling sites along the PCA axes reflected spatial and seasonal differences in environmental conditions. These variations were consistent with changes observed in phytoplankton and zooplankton abundance and composition, indicating that physicochemical factors and nutrient availability play important roles in shaping plankton community structure. High chlorophyll-a concentrations are generally associated with nutrient enrichment and increased eutrophic conditions, whereas lower concentrations indicate relatively better environmental quality^37^. Overall, the PCA analysis demonstrated that anthropogenic inputs and nutrient enrichment were the major factors controlling water quality variability and biological responses in the studied canal.

## Conclusion

In the Al-Beheira and Alexandria governorates, the Al-Mahmoudia Canal is a crucial freshwater source currently threatened by pollution from urban, industrial, and agricultural activities. This study explores the relationship between water quality parameters and plankton communities through seasonal sampling at seven stations. Findings indicate significant seasonal variations, with phytoplankton peaking in spring and winter and zooplankton affected by organic pollution during summer and fall. Heavy metals increased during the fall and spring, linked to anthropogenic activities. Multivariate analysis confirmed that water quality variations are primarily due to human inputs like agricultural runoff. The study suggests greater zooplankton diversity upstream, with identified pollution hotspots requiring targeted management. It advocates for continuous monitoring of plankton as bioindicators to support sustainable water management in Egypt’s Nile Delta.

### Recommendation

A comparative analysis against national and international regulatory thresholds, including Egyptian standards, WHO, and FAO, is needed as a next step in this monitoring study. A future publication will focus on assessing ecological and health risks according to these criteria. Furthermore, utilizing integrated environmental indices such as the Water Quality Index (WQI), Heavy Metal Pollution Index (HPI), and Metal Index (MI) will be the primary focus of an upcoming study to provide a comprehensive, modeled ecological risk assessment of the canal.

## Supplementary Information

Supplementary Information 1.

Supplementary Information 2.

Supplementary Information 3.

## Data Availability

All data generated or analysed during this study are included in this published article and its supplementary information files.
