# Supplementary figures and images for: Impacts of anthropogenic and environmental stressors on biotic communities in Al-Mahmoudia Canal, Egypt: a seasonal assessment of water quality and plankton dynamics
Source: Sci Rep. 2026 Jul 27;16:23325. doi: 10.1038/s41598-026-62334-4 (PMC13407859; doi:10.1038/s41598-026-62334-4)

**Summer**

**
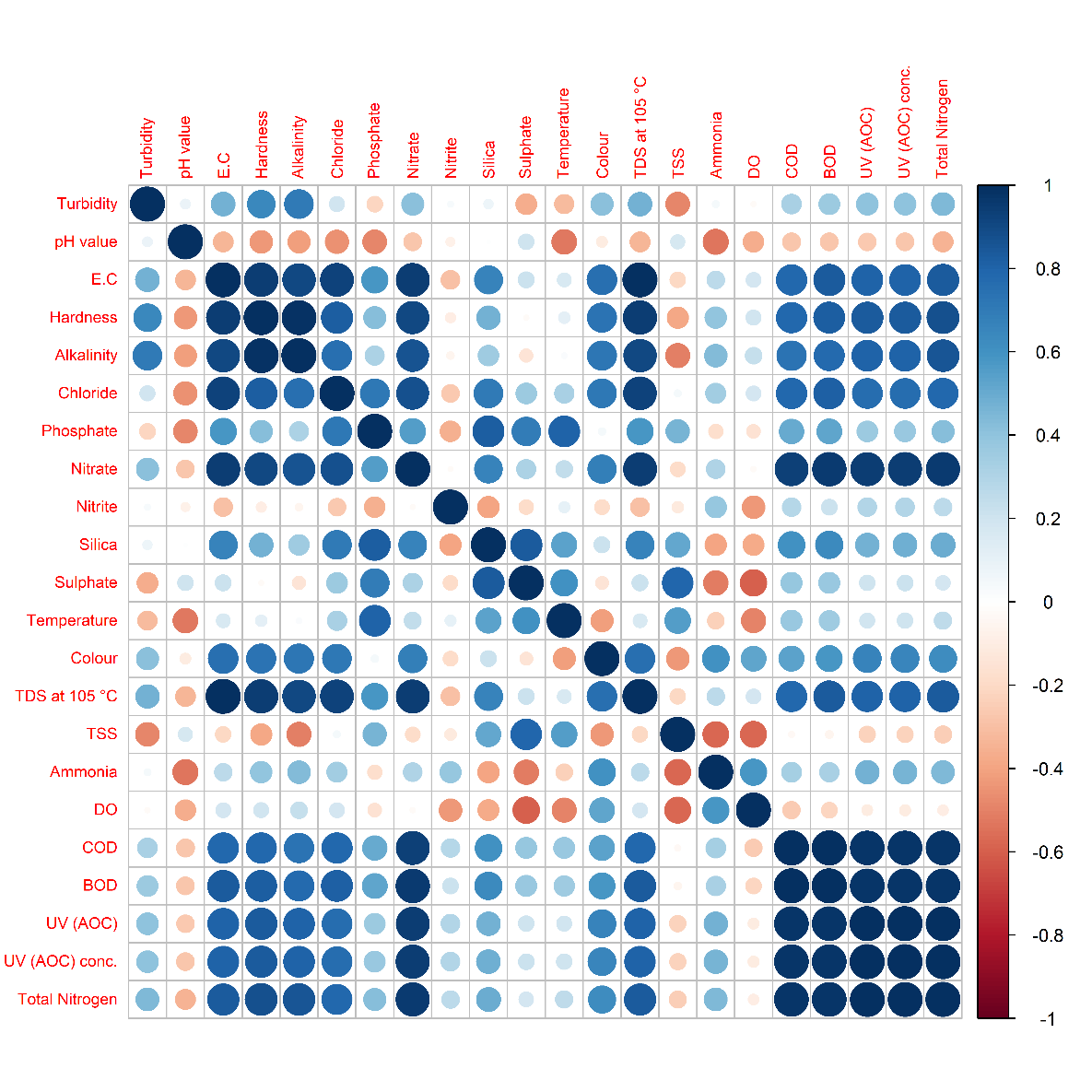
**

**
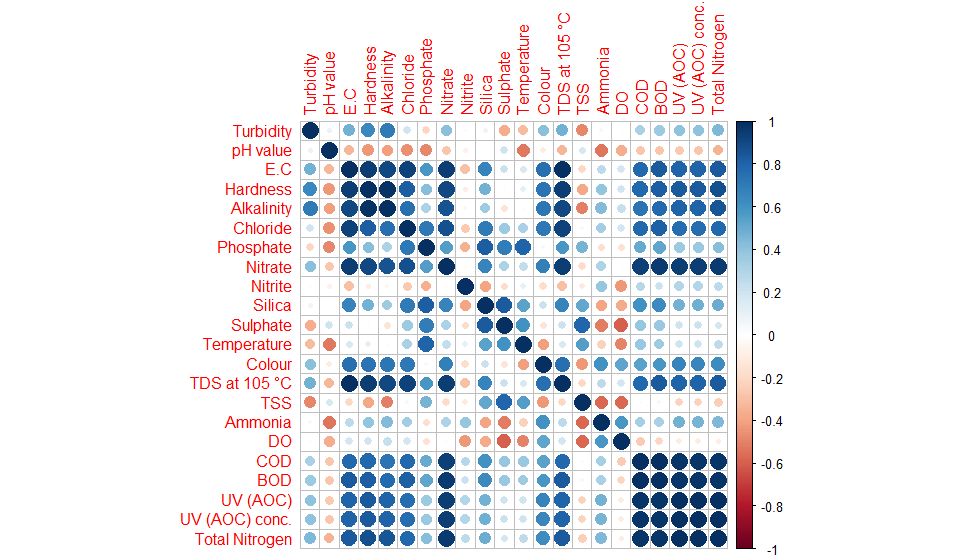
**


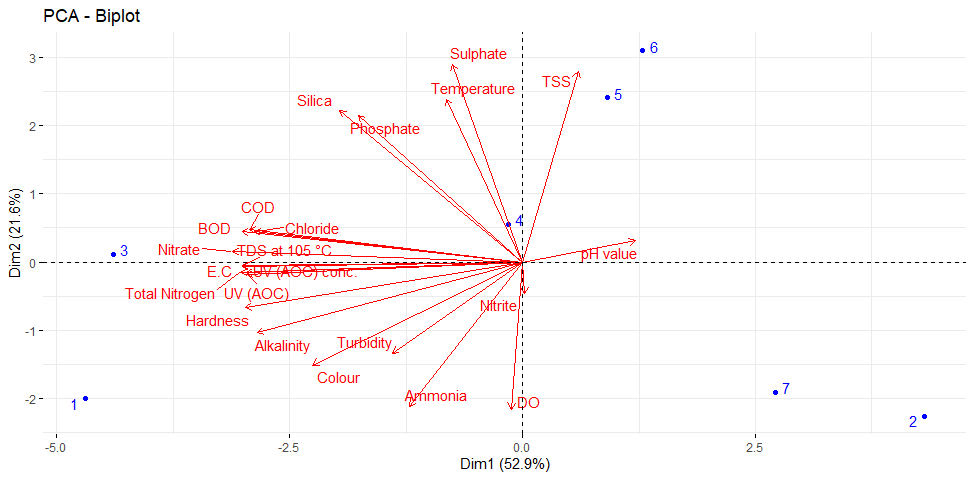


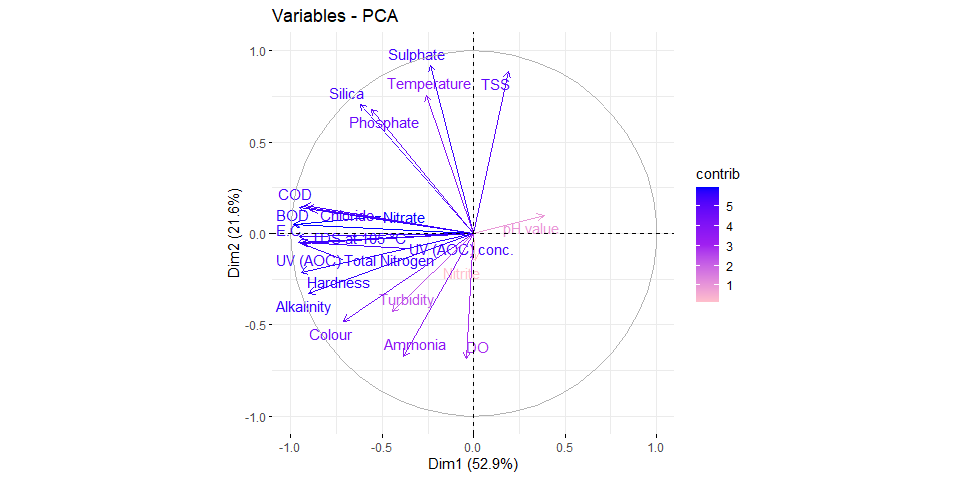


**AUTUMN**

**
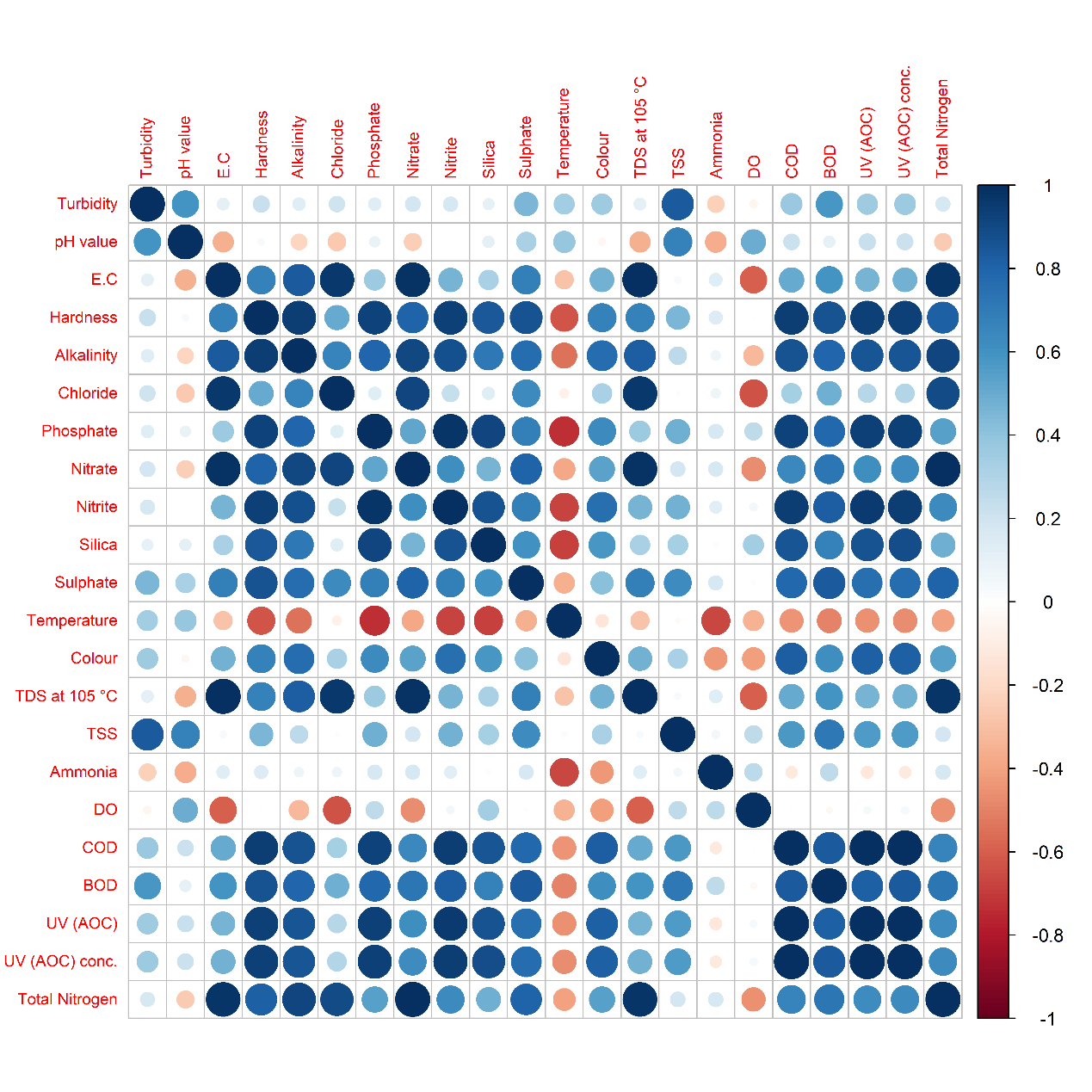
**


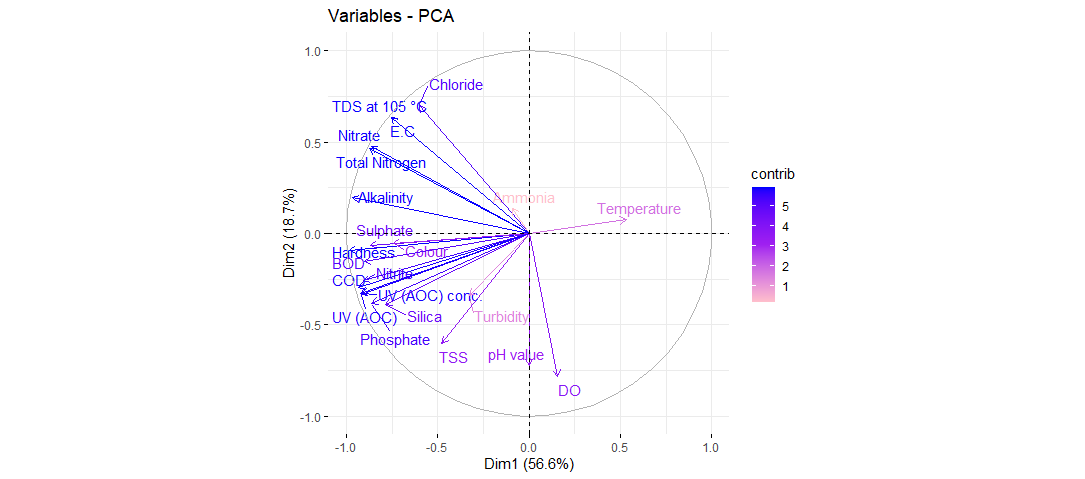


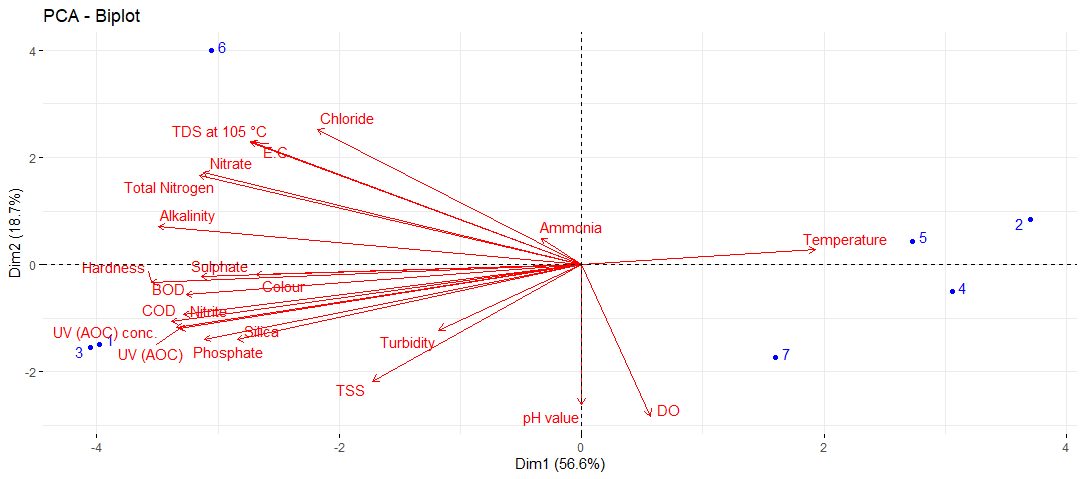


**Spring**

**
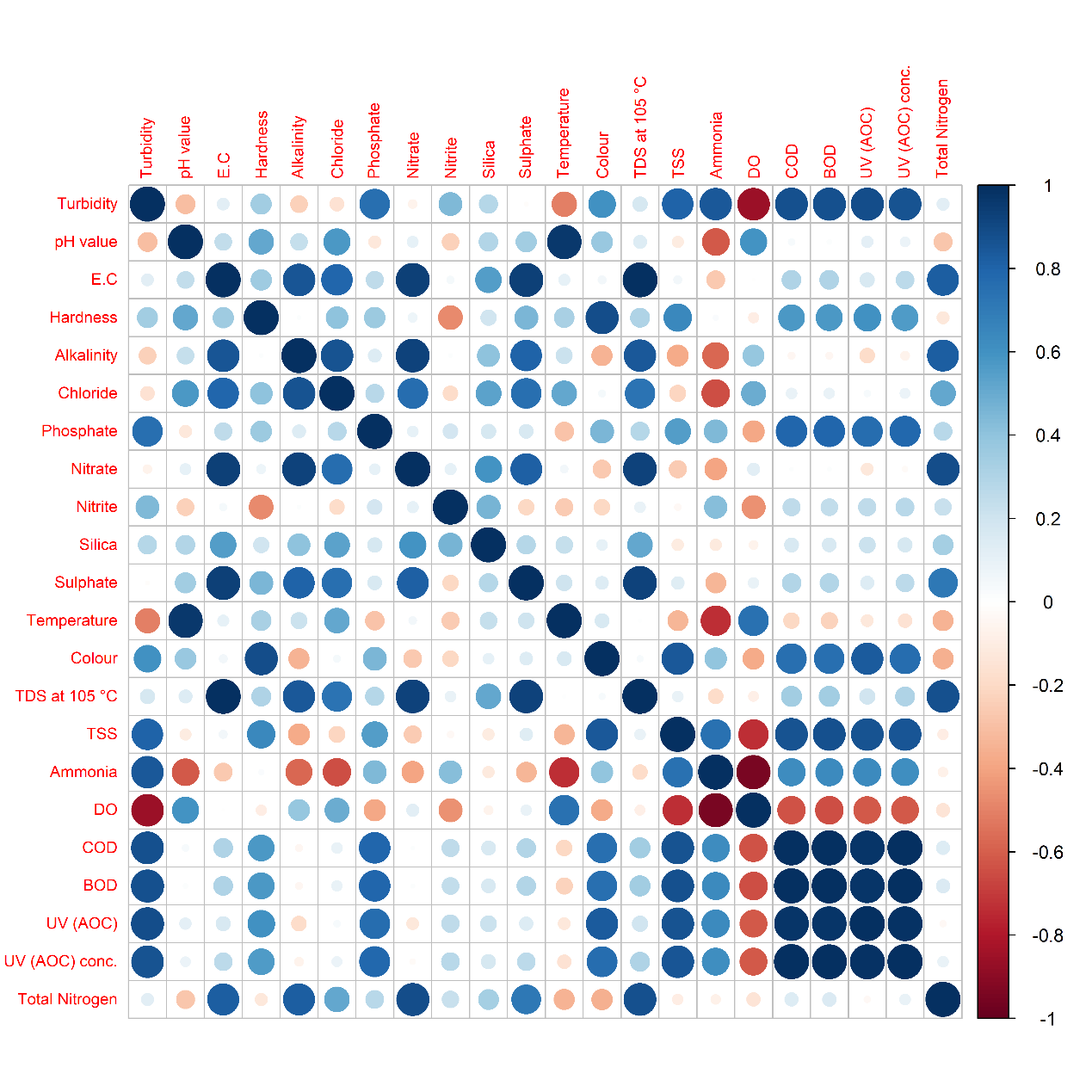
**


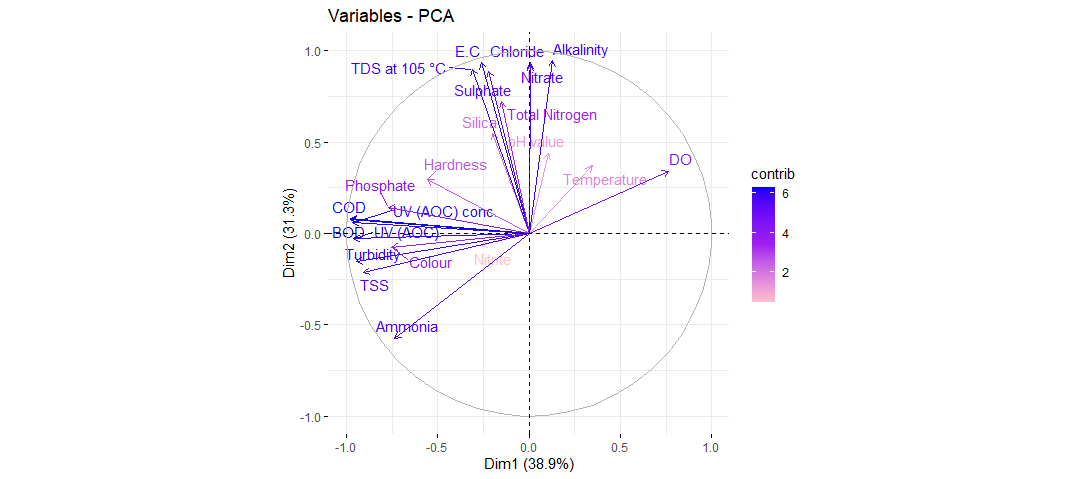


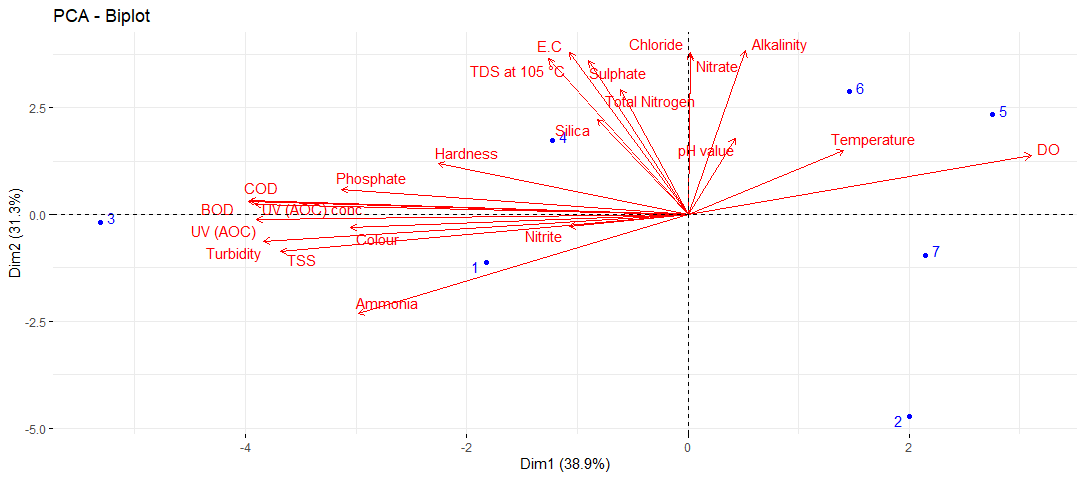


**Winter**

**
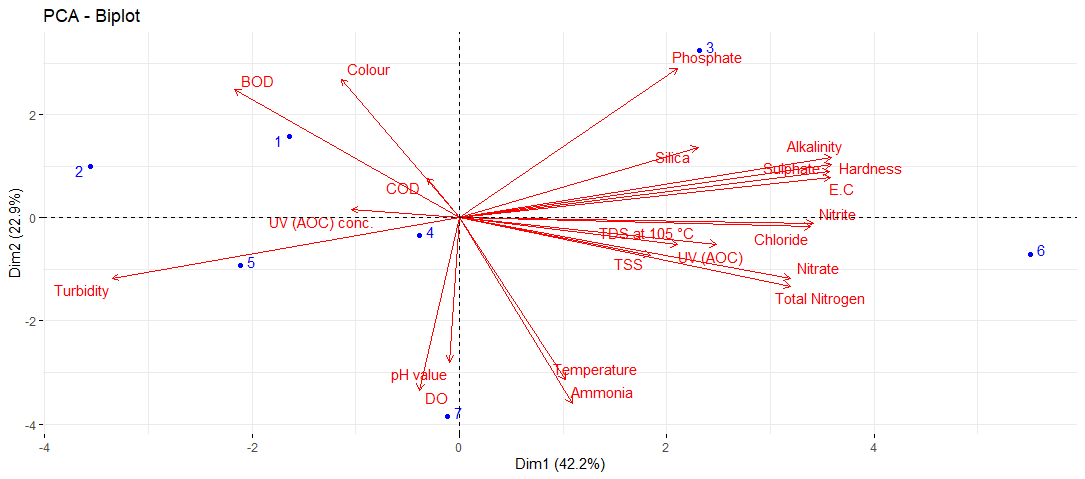

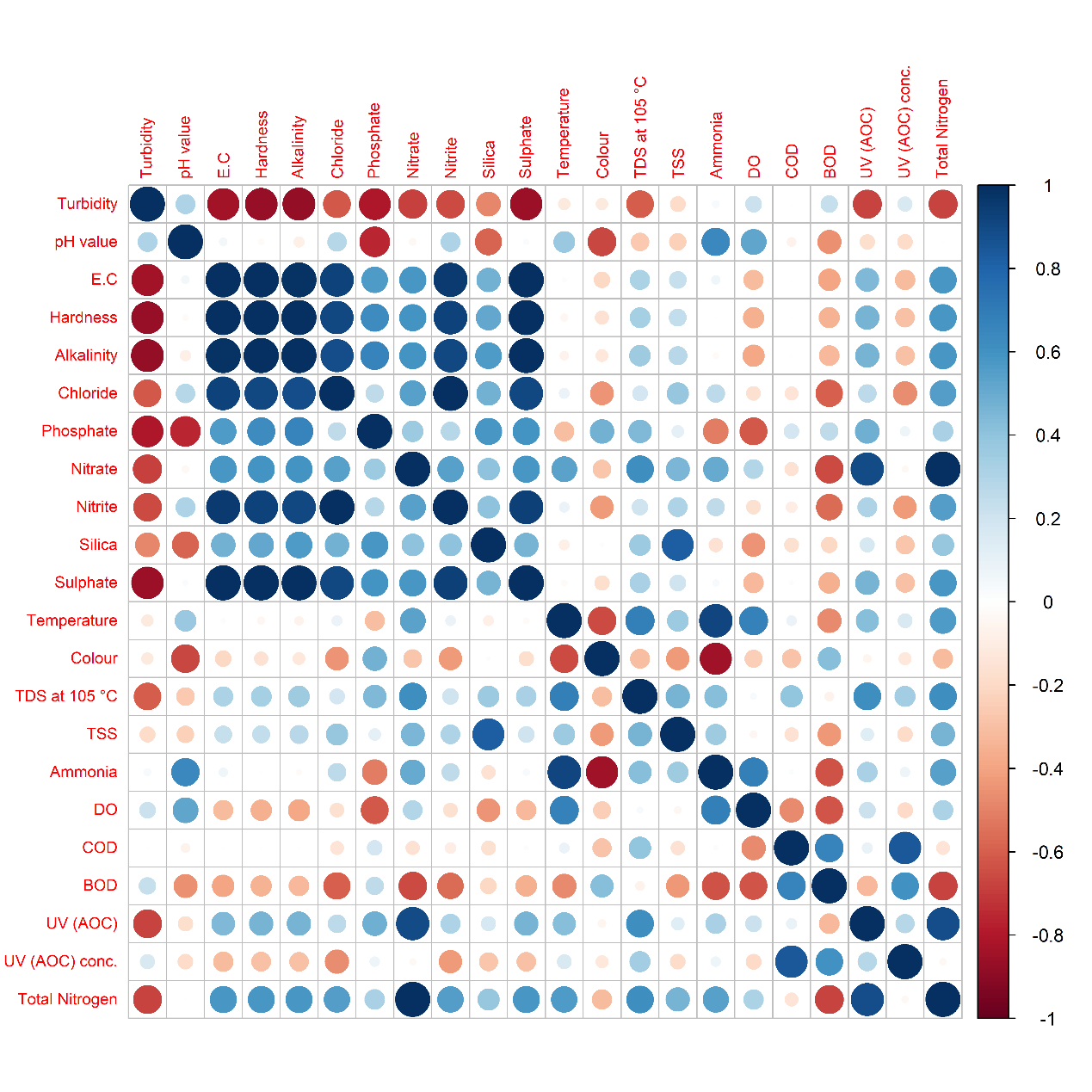
**


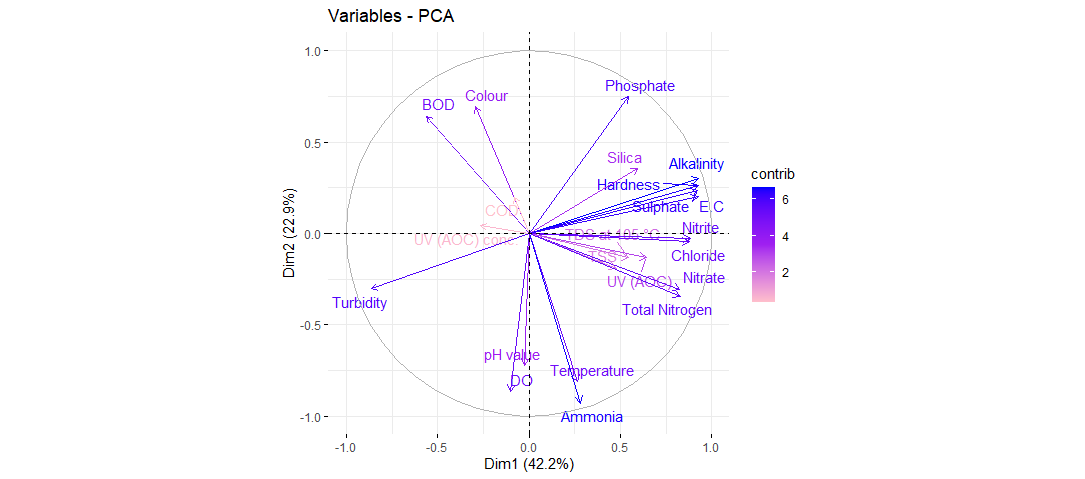


**All seasons**

**
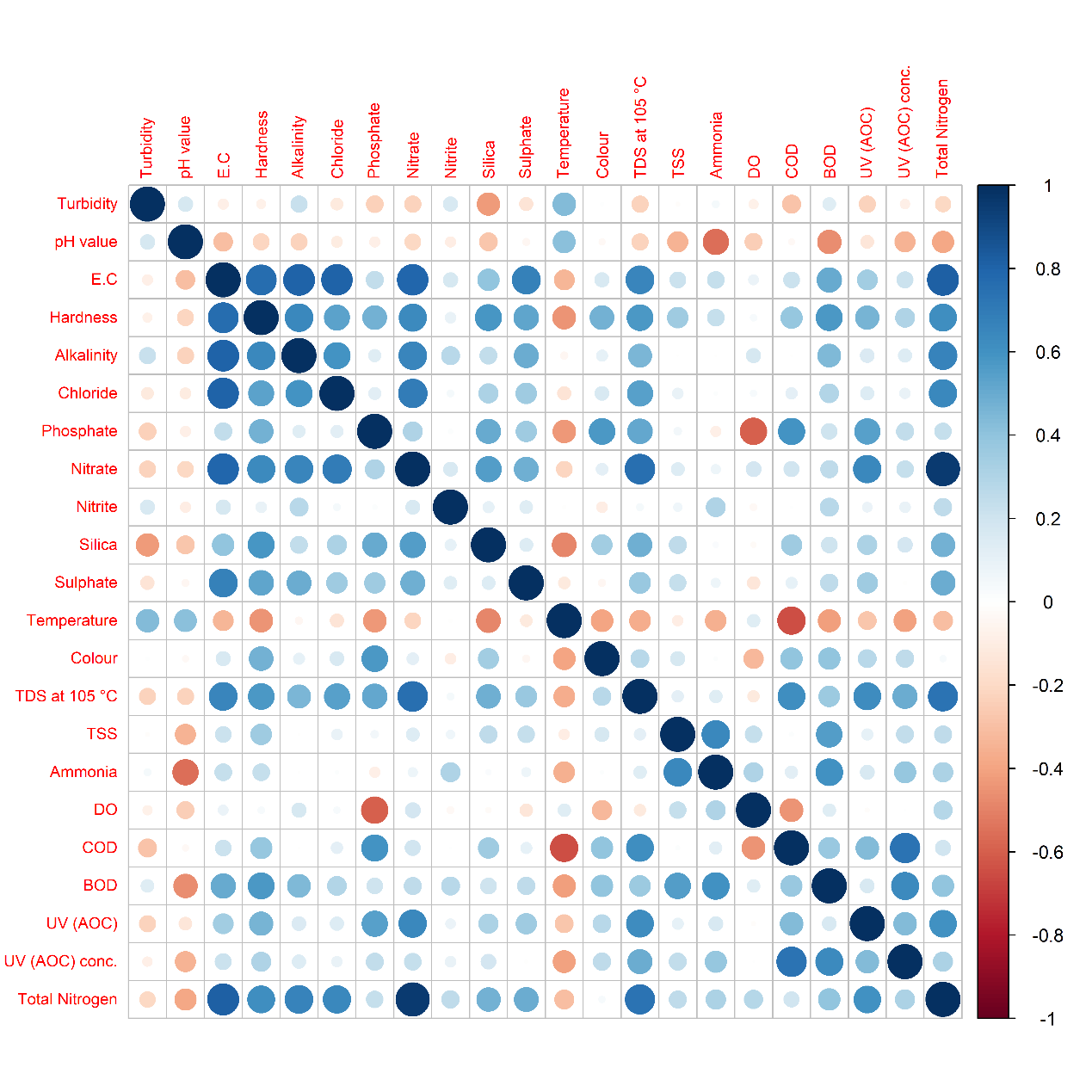
**


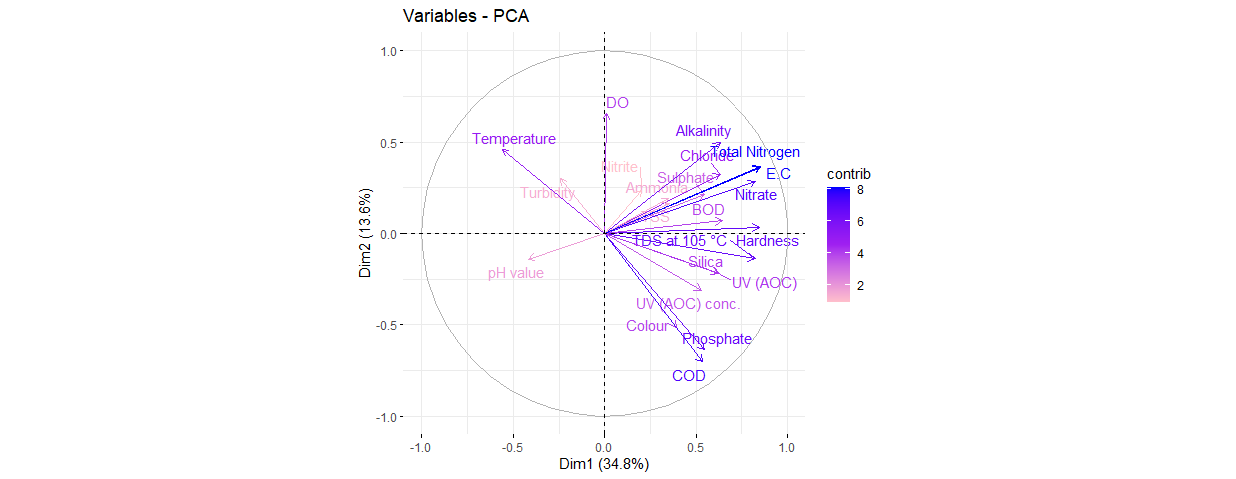


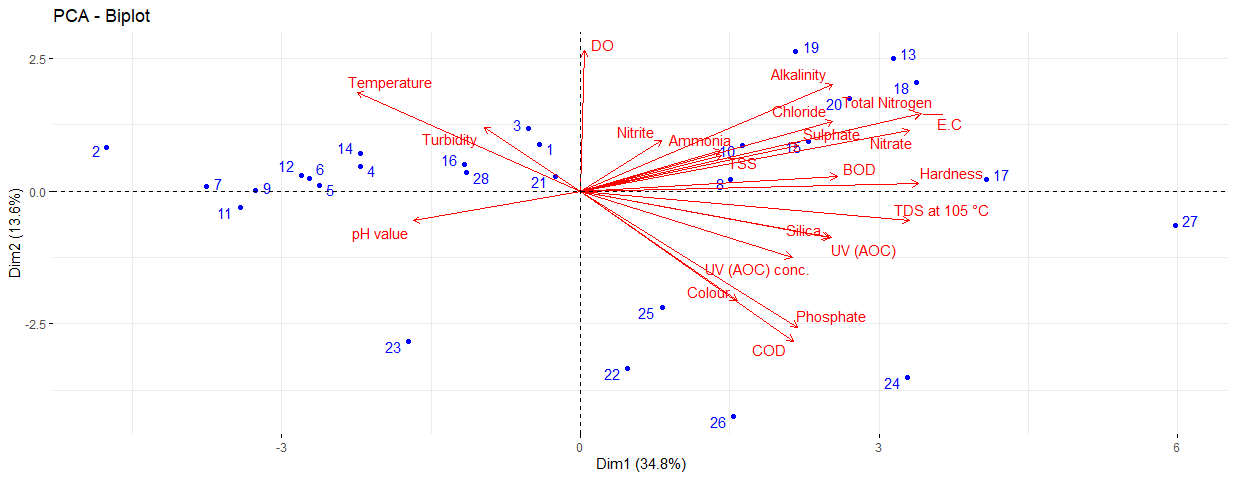


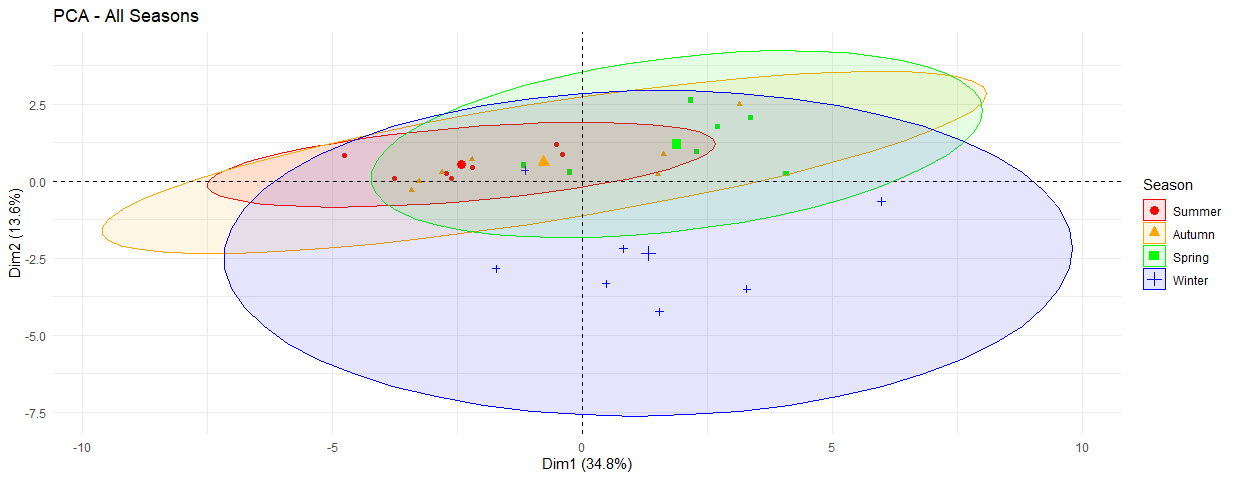


**Heavy metals**

**
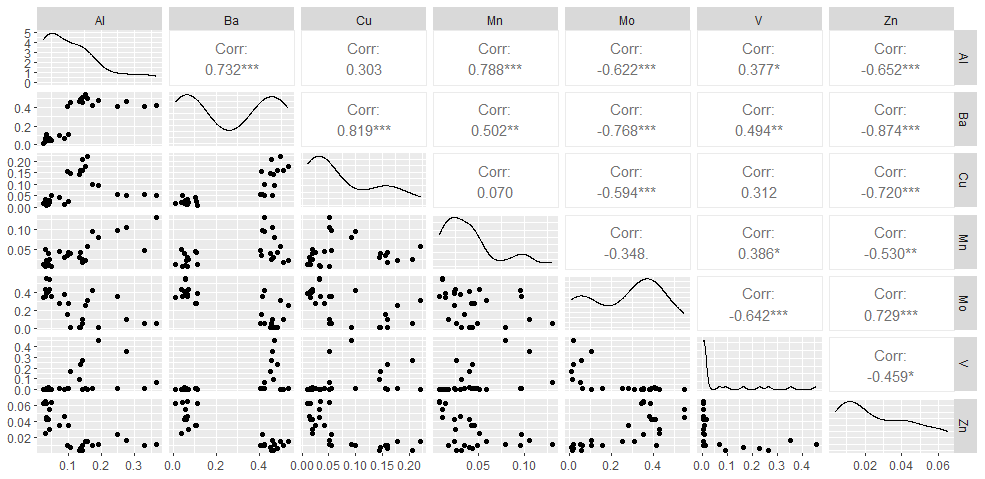
**


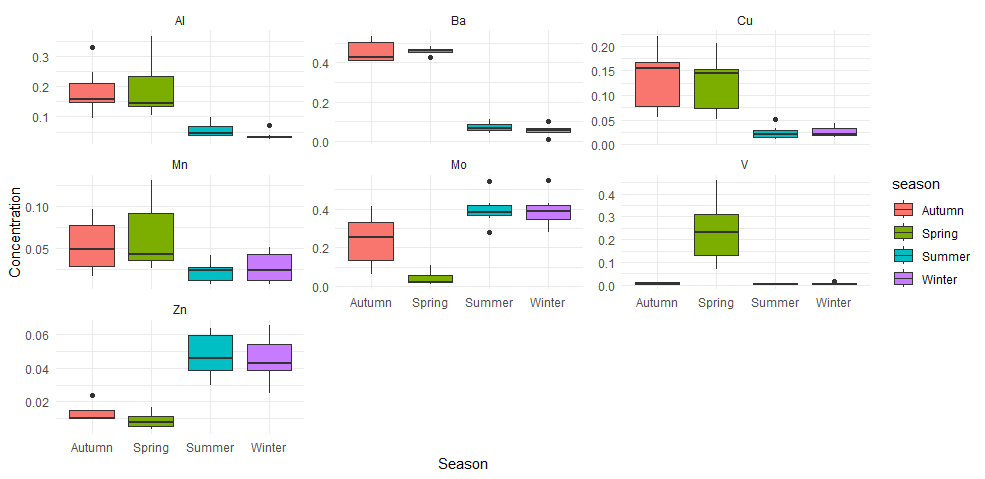


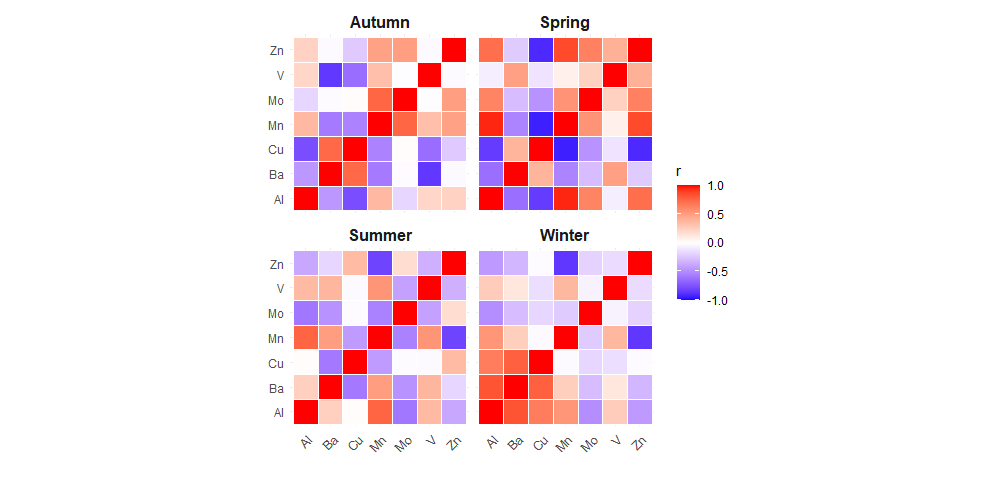

Supplement: Supplementary file 1 — Supplementary Information 1. [file 41598_2026_62334_MOESM1_ESM.docx]

**Summer**

**
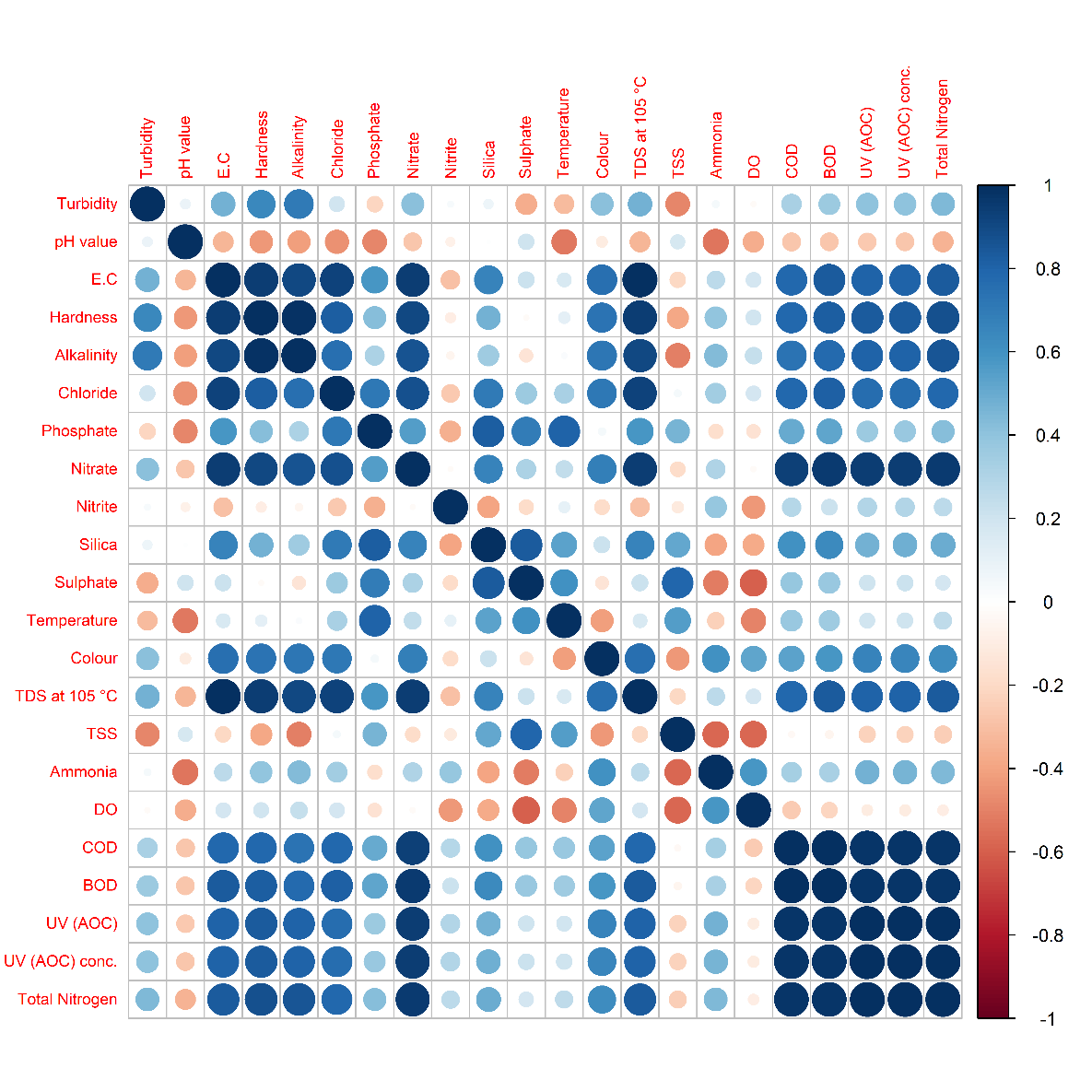
**

**
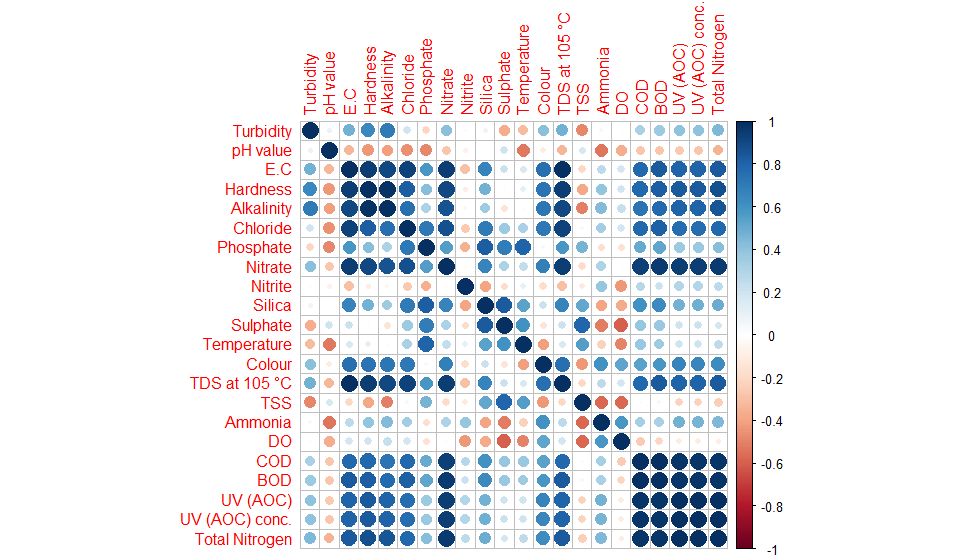
**


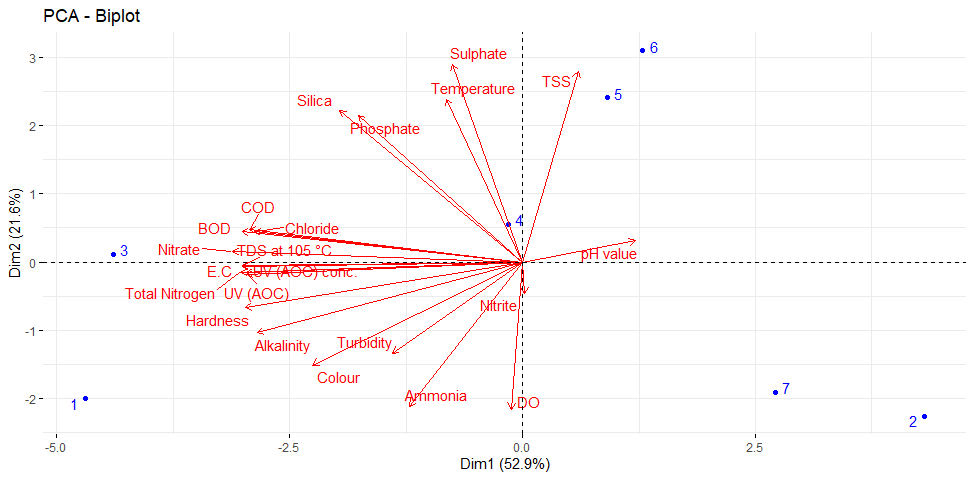


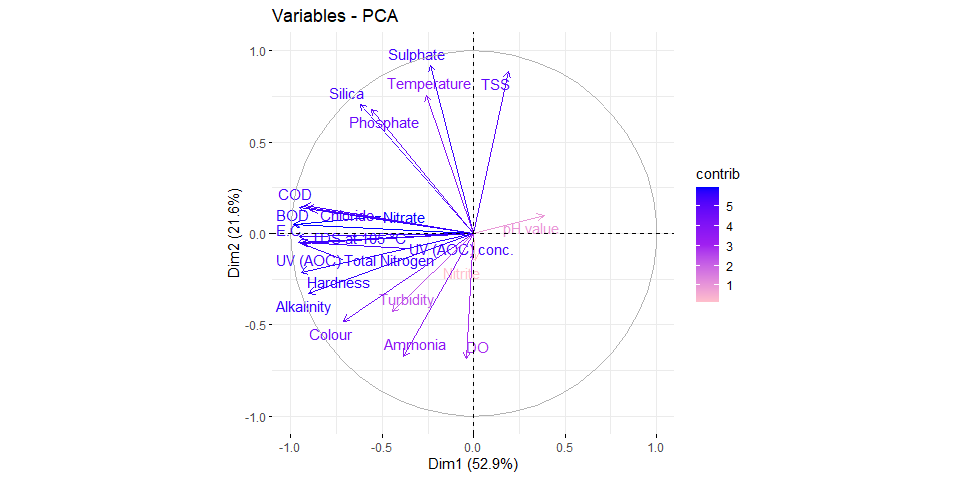


**AUTUMN**

**
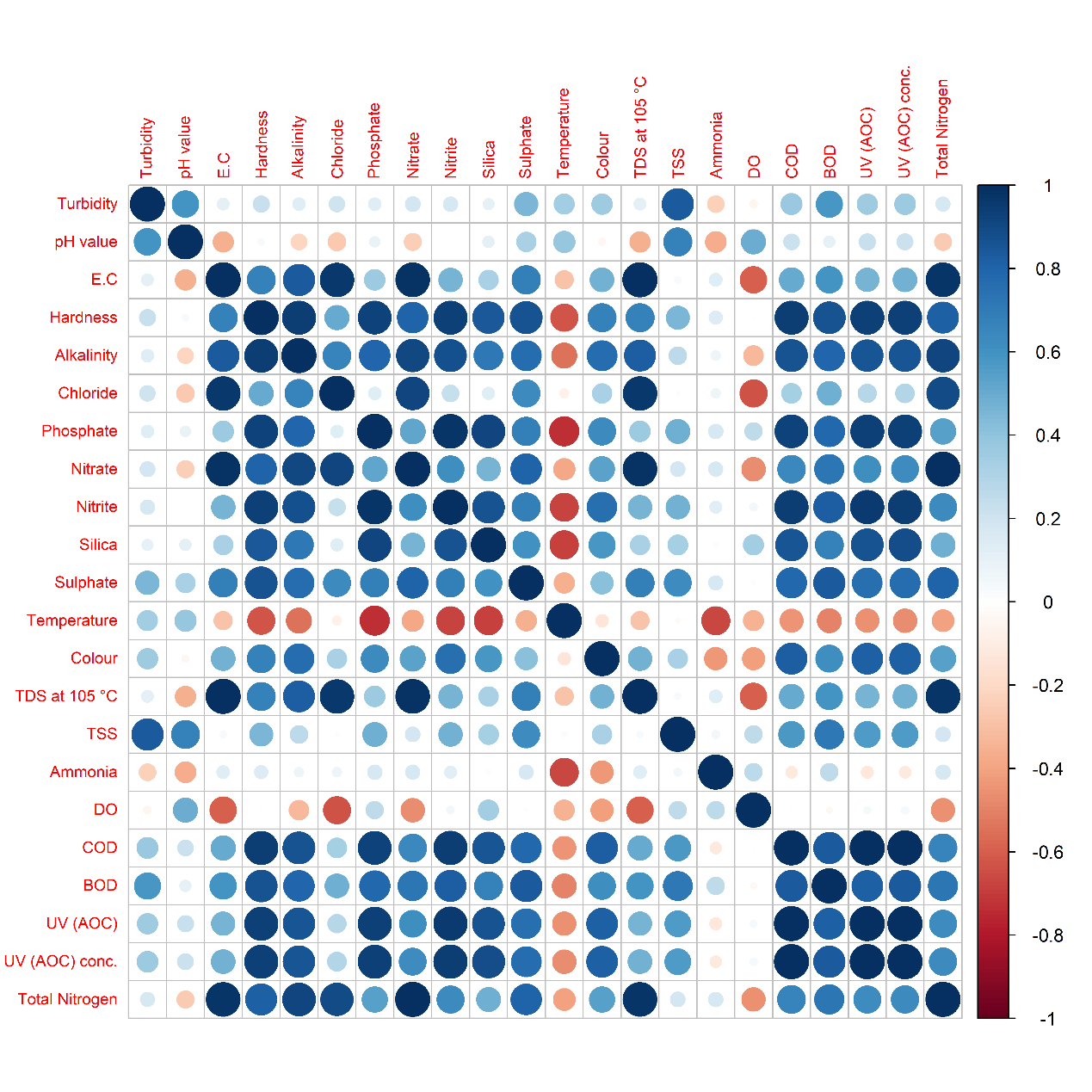
**


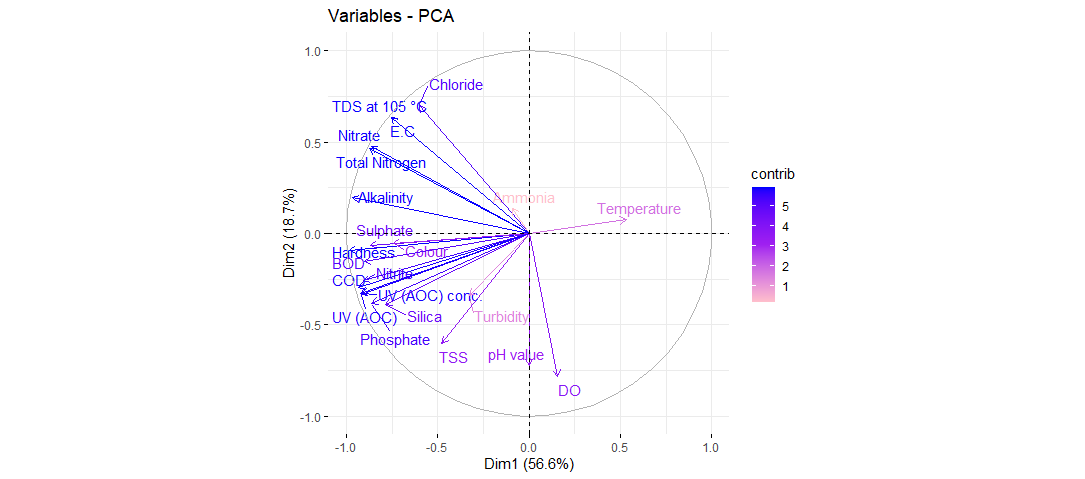


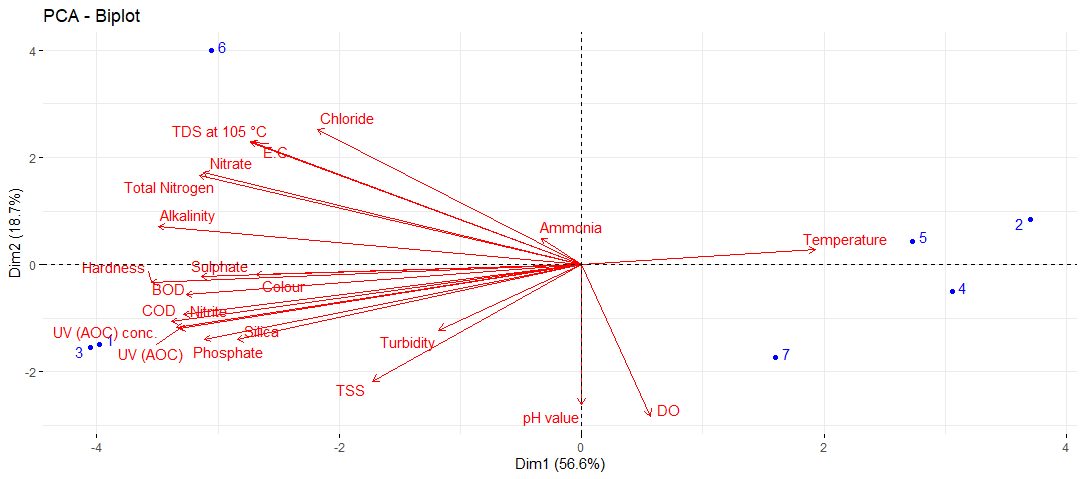


**Spring**

**
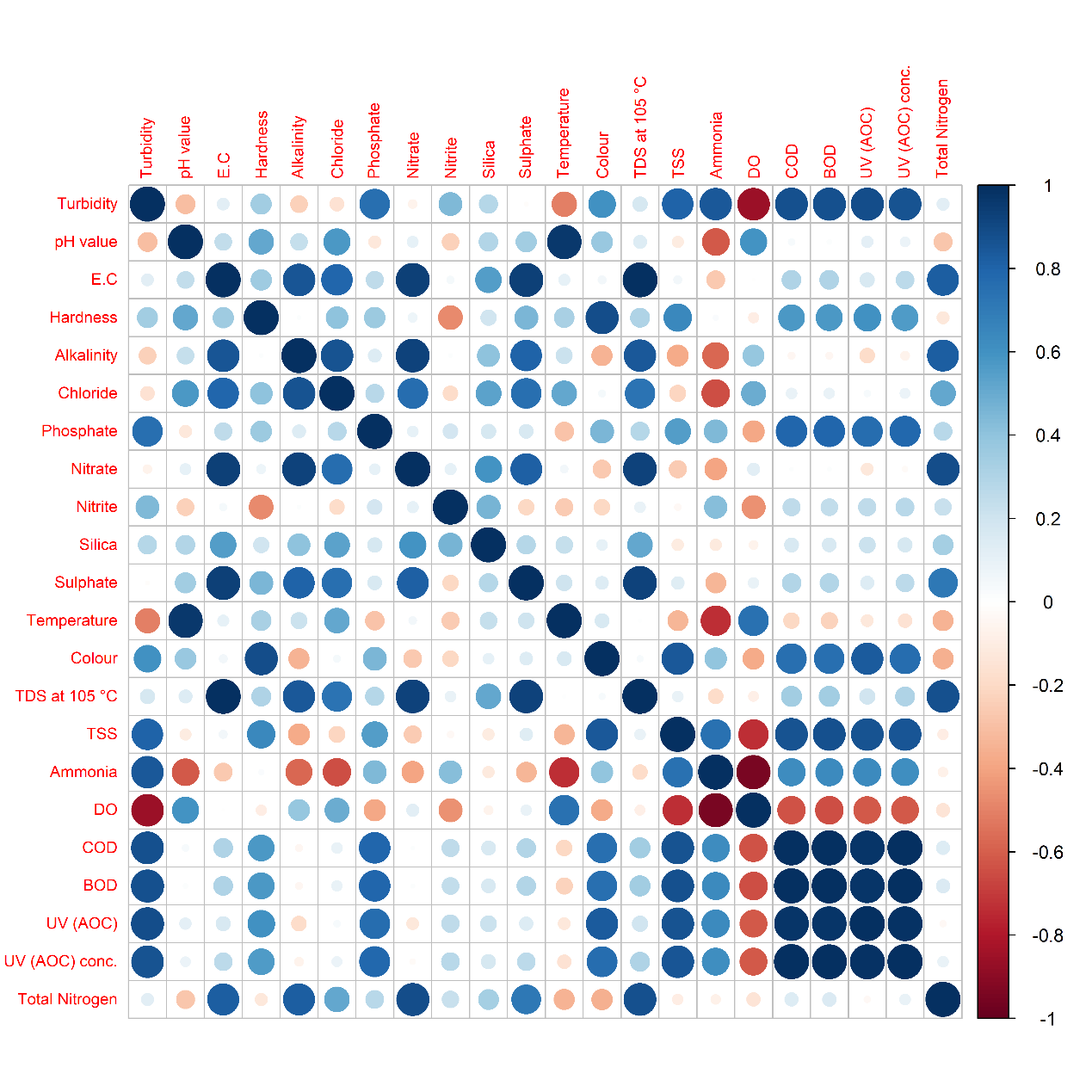
**


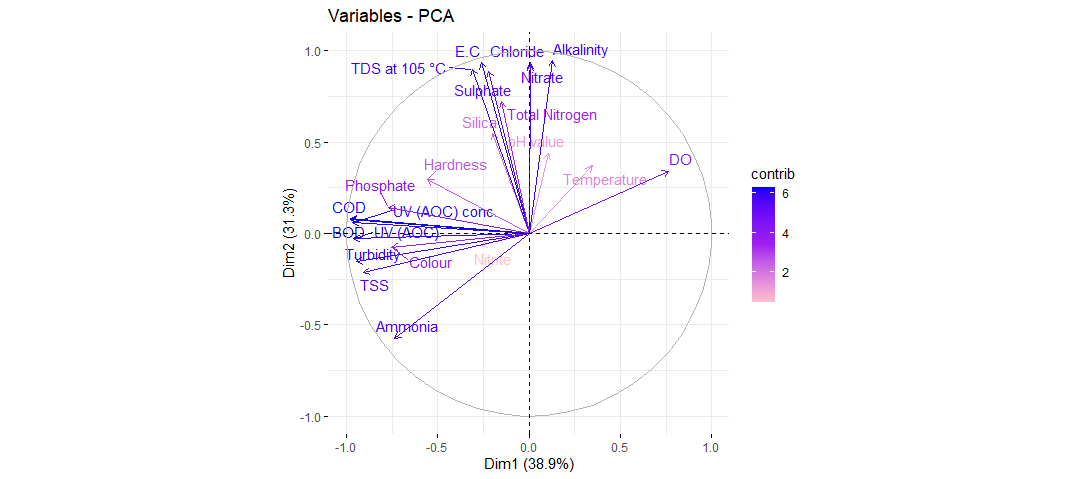


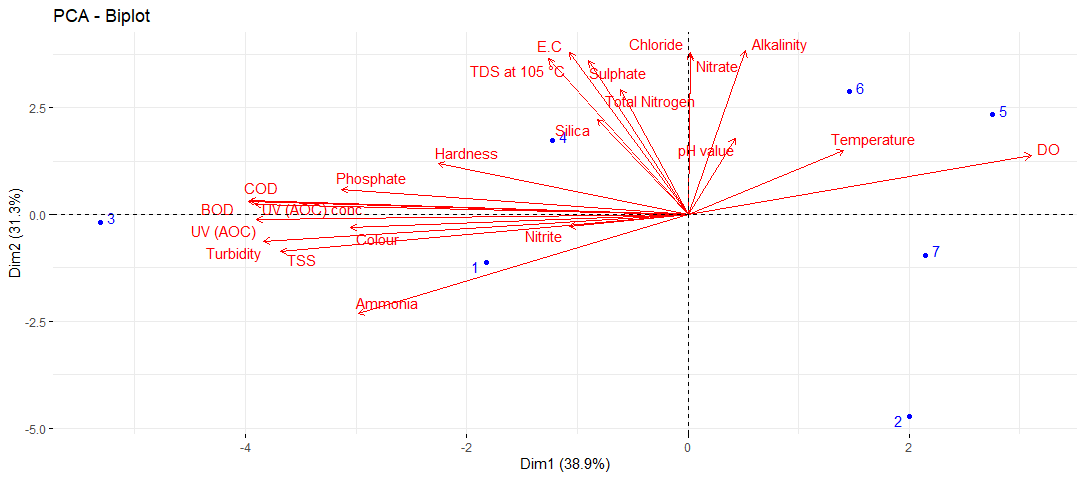


**Winter**

**
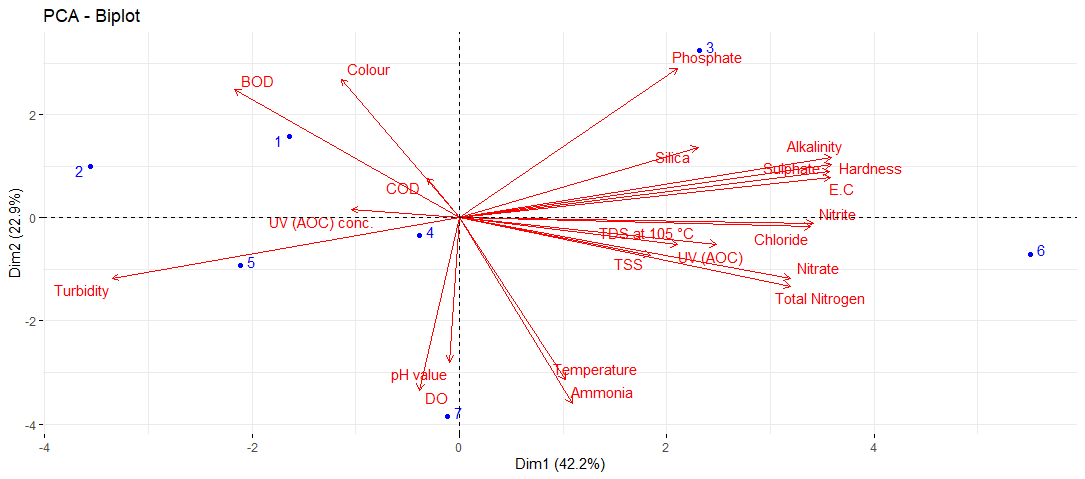

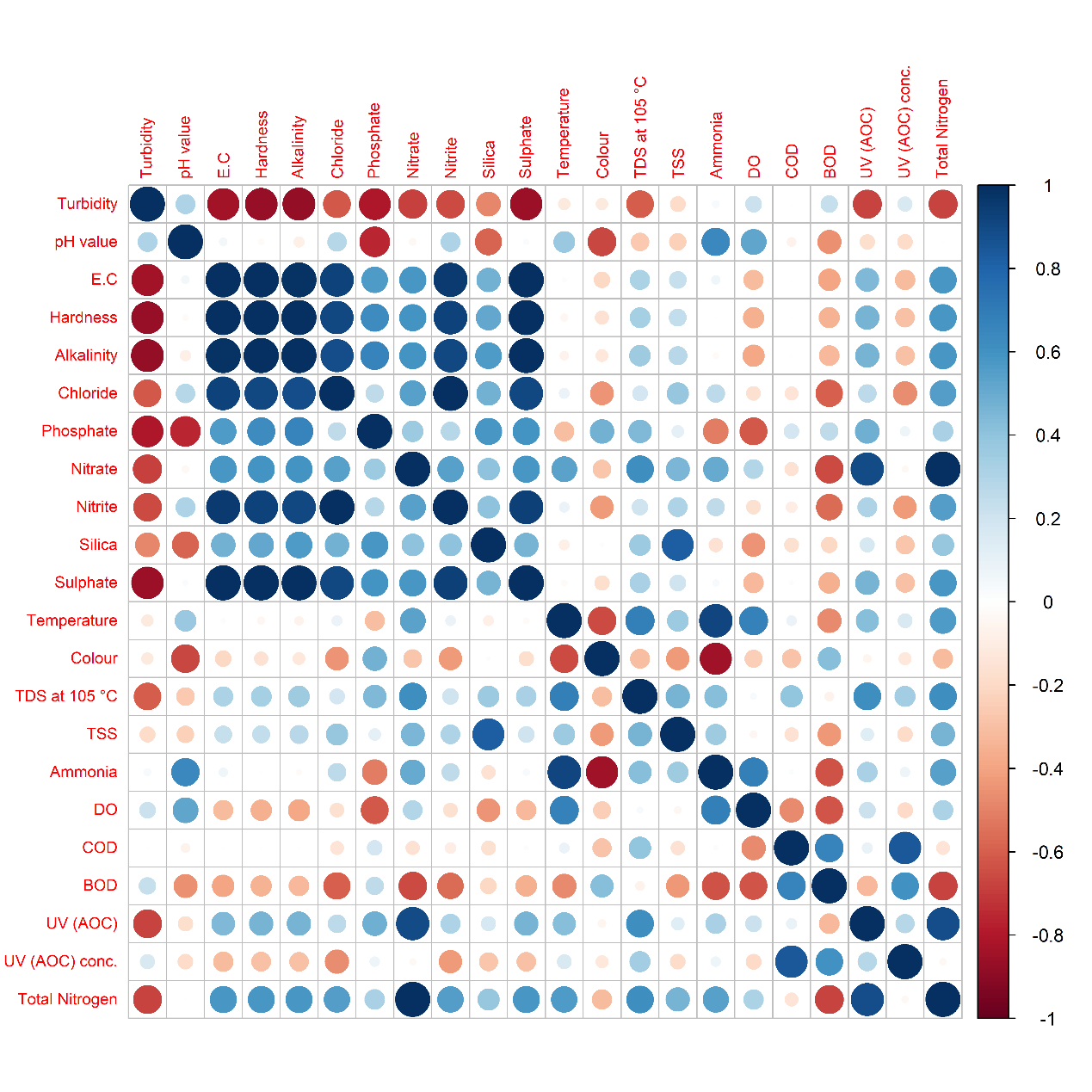
**


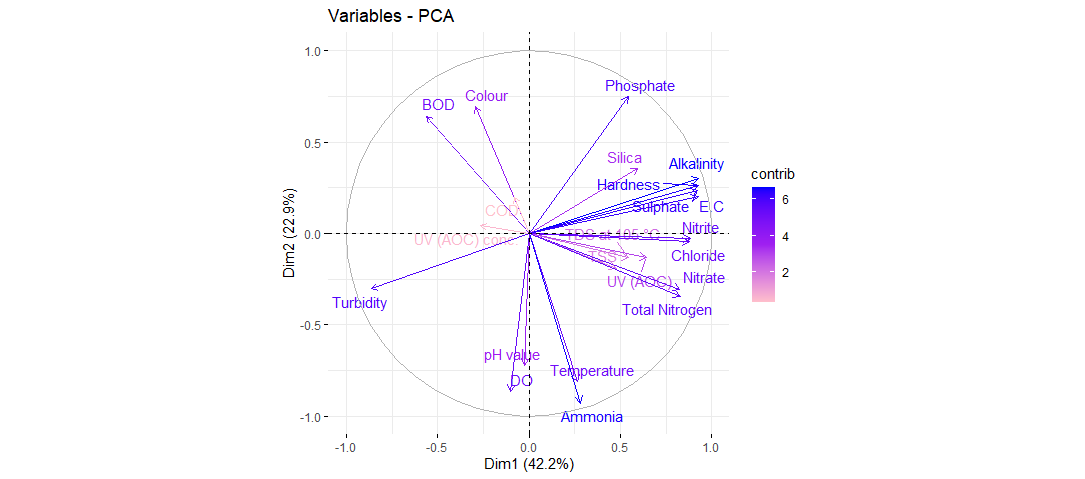


**All seasons**

**
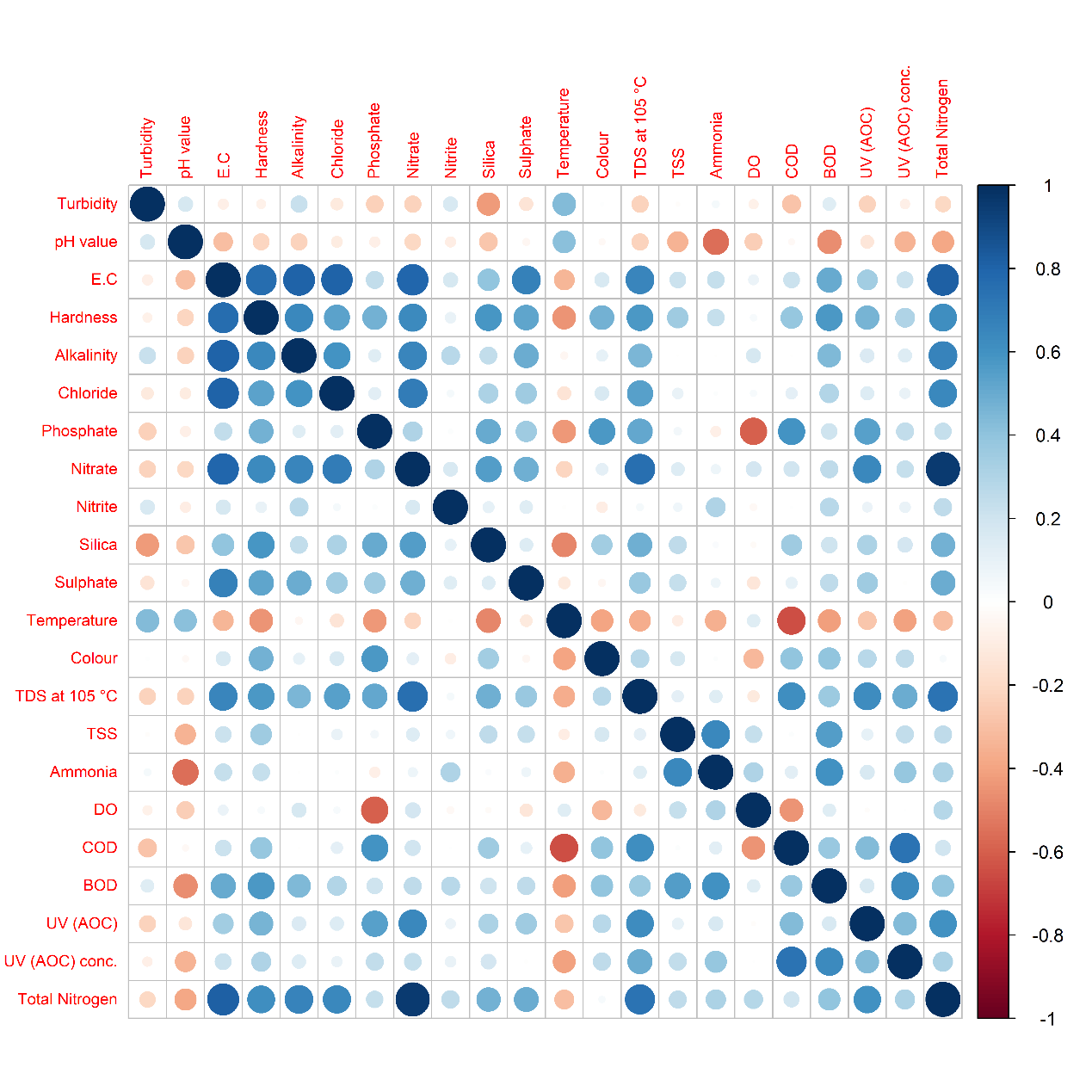
**


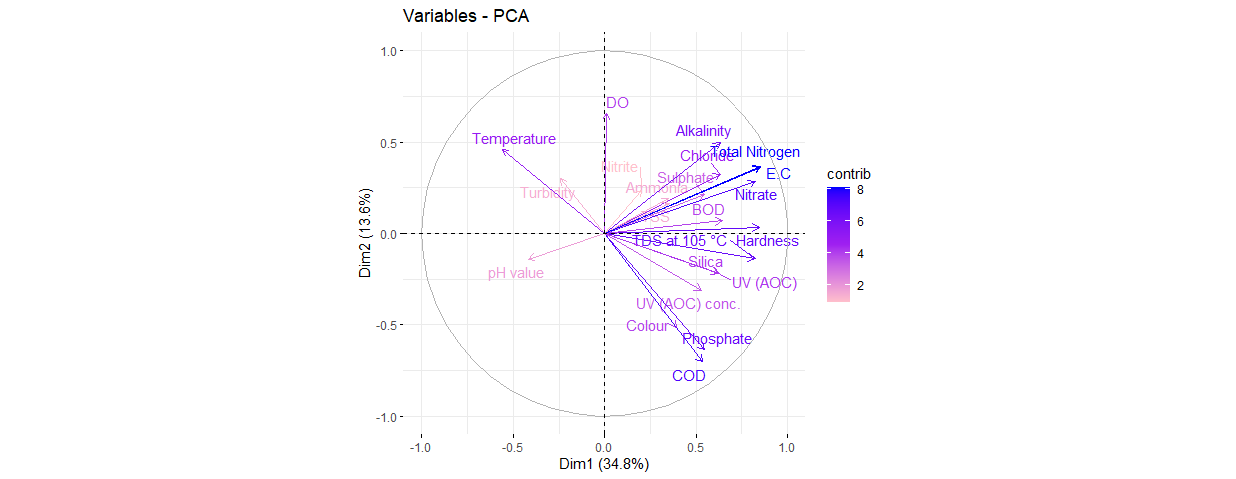


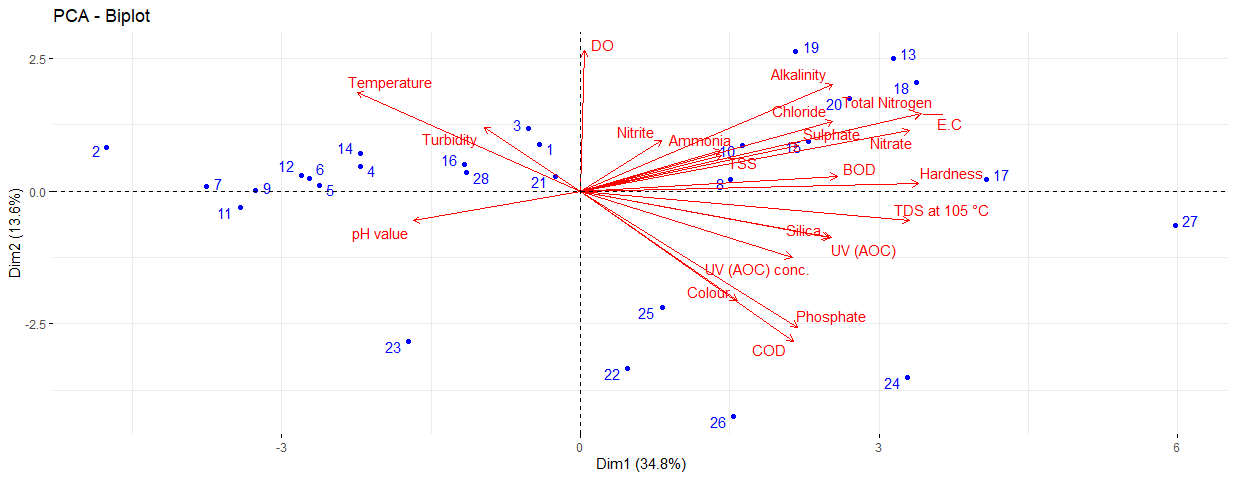


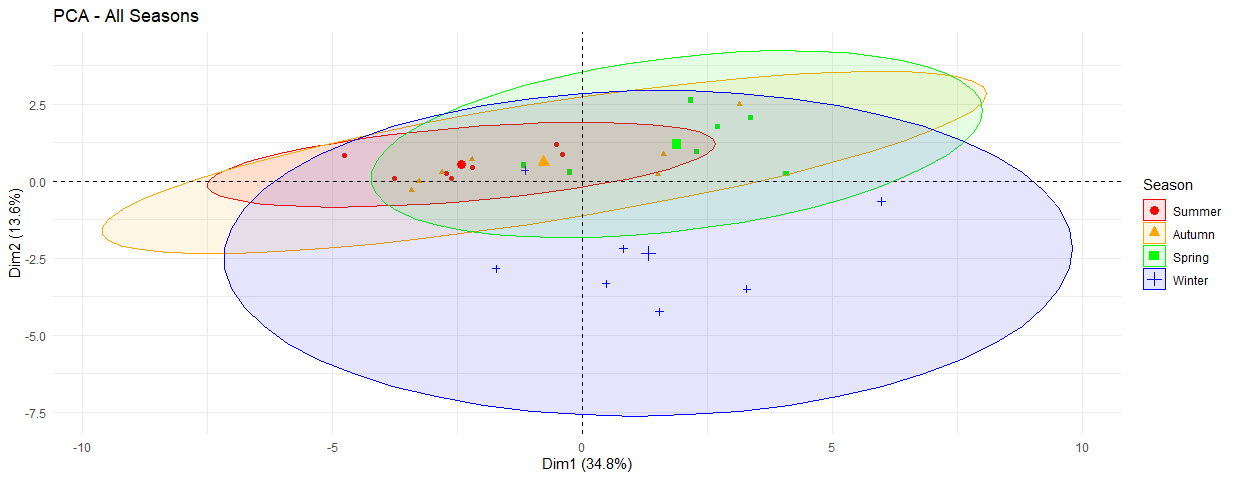


**Heavy metals**

**
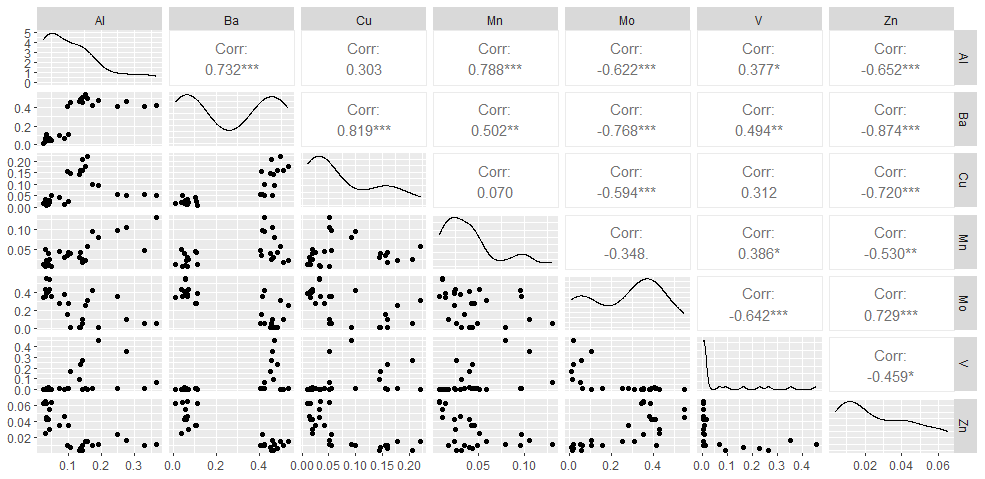
**


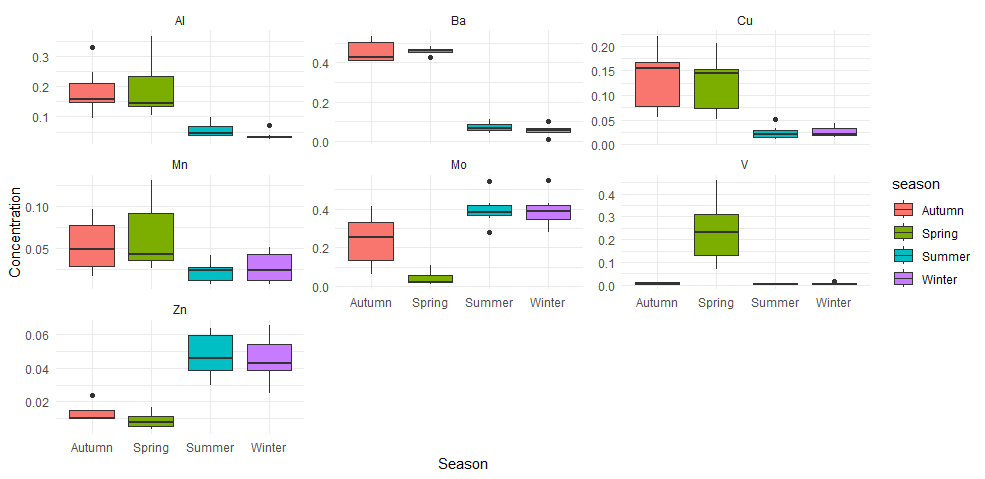


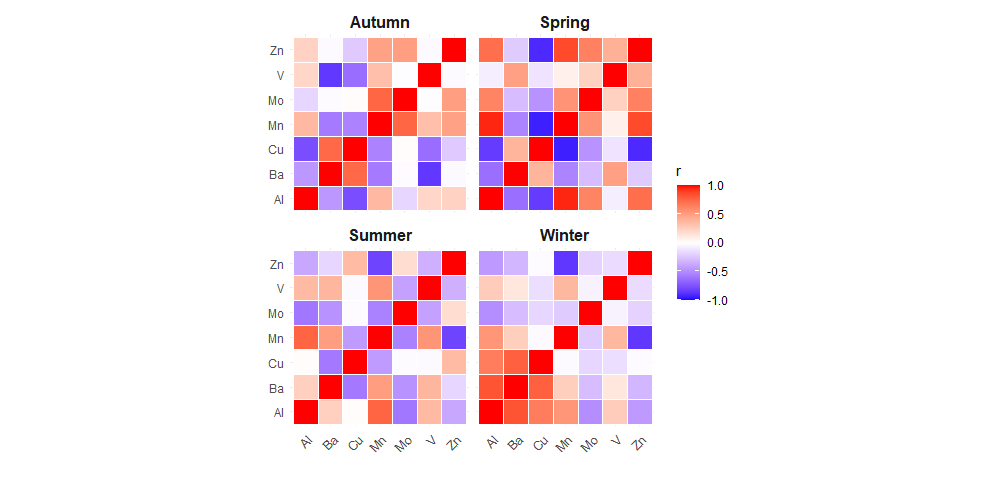

Supplement: Supplementary file 3 — Supplementary Information 3. [file 41598_2026_62334_MOESM3_ESM.docx]
